# Predicting the Dielectric Constant of Real Polar Liquids

**DOI:** 10.1021/acsomega.6c05495

**Published:** 2026-08-05

**Authors:** Konstantin I. Morozov

**Affiliations:** Department of Chemical Engineering, 26747 Technion − Israel Institute of Technology, Haifa 32000, Israel

## Abstract

The calculation of the permittivity of real polar liquids is one of the oldest problems, dating back to the classical works of Debye, Onsager, and Kirkwood. Despite its long history, such a calculation remains challenging. This is due to the many factors that affect permittivity, such as the geometry of the molecules, the distribution of dipole moments within the molecules, and the magnitude and orientation of these moments. Here, we show that the permittivity of many polar liquids admits a universal description. This occurs when short-range correlations in the dipole system are negligible. In this case, permittivity becomes a function of the average dipole interactions, which are described by the effective Debye parameter, *y*_eff_, related to the polarizability and dipole moments of the molecules, as well as their concentration. The explicit universal expression for permittivity, *ε*_uni_(*y*_eff_), is obtained from the third-order virial series in particle concentration, in which all terms depending on the short-range coupling parameter are eliminated. The relationship was applied to approximately one hundred polar liquids with the permittivities *ε* ≤ 30. The main difficulty in the calculations was the values of the dipole moments of the molecules, which are unambiguously determined only in the gas phase. In solution, the magnitude of a molecule’s dipole moment depends on its neighbors. In calculations, we used the values of dipole moments found experimentally, mainly in benzene solutions. Overall, the theoretical and experimental results for permittivity are in good agreement with each other. Furthermore, the dipole moment predictions obtained from the universal dependence *ε*_uni_ and the experimental permittivity values with high accuracy (±0.1 D) approximate the experimental moment values. Finally, the universal dependence *ε*_uni_ is generalized to the case of mixtures of polar liquids under the assumption of additivity of the mixture’s Debye parameter and volume. This generalization provides an accurate lower estimate of the permittivity of a mixture of real polar liquids.

## Graphical abstract

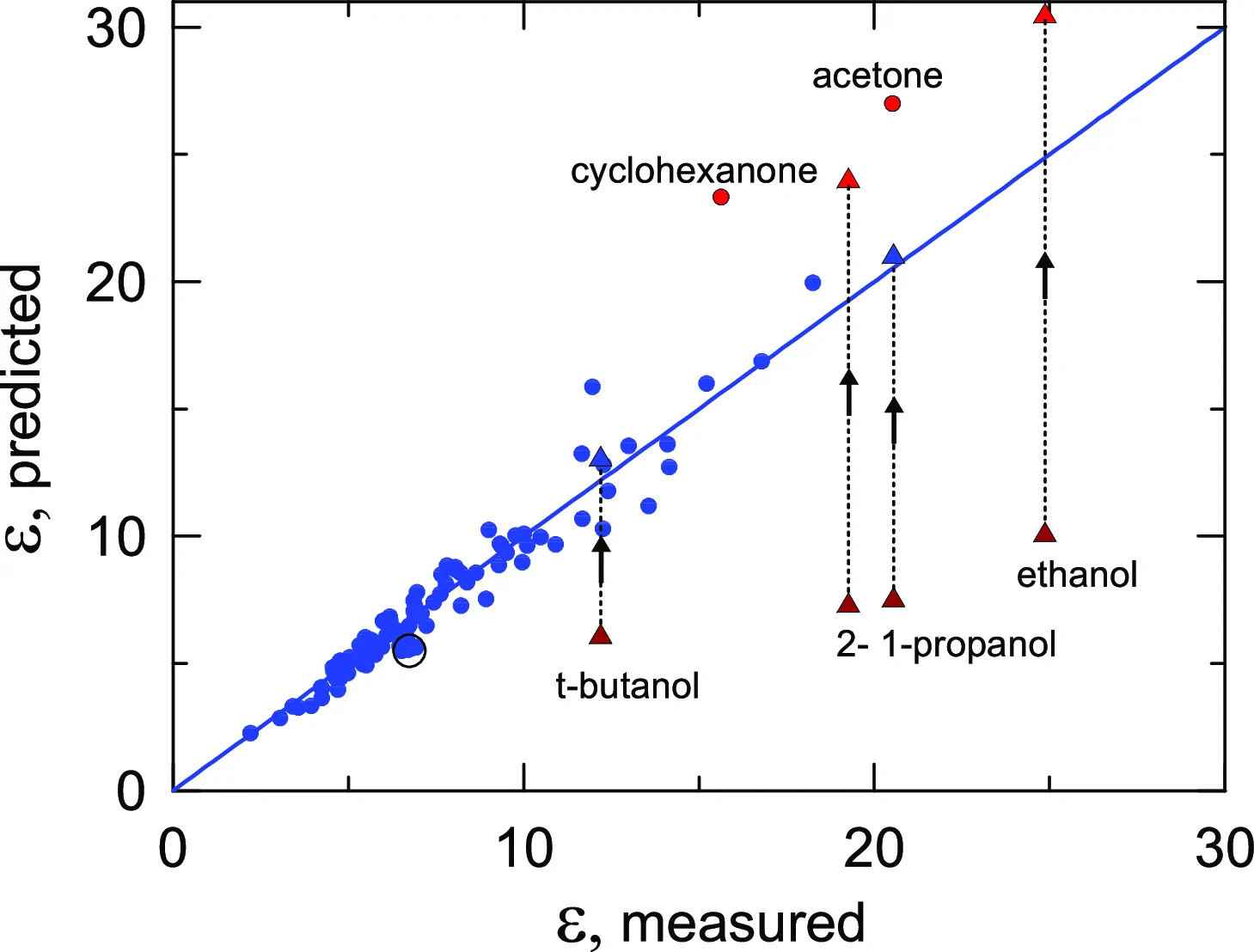

## Introduction

Real polar liquids differ significantly from each other in terms of their molecular geometry, the distribution of dipole moments within their molecules, and the magnitude and orientation of these moments. For highly polar substances, these values can reach 3–5 D. The main question we address here is to what extent the permittivity of these liquids can be described, and whether it is possible to provide a universal description that covers a wide range of liquids. As we will demonstrate below, the path to real polar liquids passes through two other systems. The first type comprises magnetic fluids, ferrocolloids or ferrofluids—artificial systems of magnetic dipoles dissolved in a liquid matrix.^1,2^ The second type is a purely computational system that models the polar liquid as an ensemble of hard sphere (HS) dipoles.^3^

Ferrofluids became the subject of detailed research in the 1960s and have since been well characterized. Their thermodynamic stability and behavior in external magnetic fields have been studied. In particular, magnetization curves and initial susceptibility in static and periodic magnetic fields have been investigated theoretically,^4–6^ experimentally^7,8^ and numerically.^9,10^

Compared to real polar liquids, ferrofluids have several advantages inherent to artificial systems. First, they have *tunable* magnetic properties. Magnetic nanoparticles (∼10 nm) are single-domain^1,2^ with a magnetic moment *m* = *M*_s_ *v*, where *M*_*s*_ is the magnetic saturation magnetization and *v* is the particle volume. Thus, the magnetic moments of the particles can be easily varied by selecting the appropriate magnetic material and particle size. In practice, ferrofluids based on iron oxides, cobalt and nickel are most often used. Second, magnetic nanoparticles are usually spherical. Typically, synthesizing nonspherical colloidal particles is associated with significant technical difficulties.^11^ Third, the amount of magnetic material is also adjustable and can reach about 20% of the volume of the entire system. Finally, the interparticle interactions are accurately described by the potential of dipolar hard spheres. This makes magnetic fluids an ideal model for polar fluids. The only complicating factor in this model is the polydispersity of ferrocolloids, a property that real polar liquids do not possess. However, polydispersity is also a controllable variable. Methods for synthesizing virtually monodisperse magnetic fluids (MFs) are now known.^12^ Moreover, well-developed techniques exist for accounting for particle size distribution within a given description model.^13^ The magnetic analog of the dielectric constant *ε* of polar liquids in MF is magnetic permeability μ = 1 + 4*πχ*, where χ is the susceptibility of the MF that determines the initial slope of the magnetization curve *M* = *M*(*H*) in an external magnetic field. In what follows, we will completely ignore saturation effects and deal with the case of an infinitesimal, static external field.

A Monte Carlo (MC) simulation of the permittivity of the dipolar HS fluid was made possible by the development of the Ewald sum technique for dipolar systems.^14^ Since then, this technique has become the primary method used in all subsequent MC calculations.^10,15–20^ In ref 19 the technique was applied to polarizable dipolar hard sphere fluids. Recently, Theiss and Gross obtained comprehensive MC simulation data on the permittivity of dipolar hard sphere fluids.^21^ Their results are the most accurate to date and will play a key role in the present study.

In the following consideration, we will first describe the known analytical models of magnetic fluid susceptibility/permeability. Then, we will discuss their agreement with numerical calculations and their relationship with real polar liquids.

## Methods

### Models of the Initial Susceptibility of Magnetic Fluid

We consider MF as an ensemble of hard spherical particles with the diameter *d*, permanent magnetic moment *m*, and number concentration *n*. In the simplest approximation of noninteracting particles, when orientation of each dipole is determined by competition of magnetic torque and thermal agitation *k*_*B*_ *T*, the magnetic susceptibility χ of the system reduces to its Langevin value χ_*L*_ = *nm* ^2^/(3*k*_*B*_ *T*). Correspondingly, the magnetic permeability μ becomes

1 $$\mu_{L}=1+4\pi nm^{2}/\left(3k_{B}T\right)=1+3y$$

where we used the Debye parameter *y* = 4*πχ*_*L*_/3 = 4*πnm* ^2^/(9*k*_*B*_ *T*).

Dipole interparticle interactions create an additional field that aligns the magnetic moments of the particles, leading to an increase in magnetic susceptibility. Along with the Debye parameter *y*, (or equivalently, the Langevin susceptibility χ_*L*_) the dipole interactions can be characterized by the coupling parameter λ, defined as

2 $$\lambda =m^{2}/\left(d^{3}k_{B}T\right)$$

Physically, both parameters describe dipolar interparticle interactions. However, the Debye parameter quantifies the *average* dipolar interactions in the system, while the coupling parameter relates to the interaction of two particles *in contact*. In other words, the Debye parameter *y* and the coupling parameter λ describe the long- and short-range correlations in a system of dipoles, respectively. When dipole interactions become comparable to thermal energy, λ ∼ 1, the system begins forming simple structures—clusters of pairs of particles with a preferred head-to-tail orientation of magnetic moments.^22^ At 1 ≤ λ ≤ 3, more complex structures emerge in the system—short chains^23^ or small quasi-spherical clusters made of two to ten grains.^9,24^

Returning to the description of the magnetic permeability of ferrocolloids, it can be stated that, in general, it depends on both the dipole parameters −*y* and λ. However, when the coupling parameter is small, λ ≤ 1, the short-range effects are negligible. In this case, the magnetic permeability becomes a function of the average dipolar interactions only: μ = μ­(*y*). This property is realized in two classical mean-field theories of dipole systems: the Debye^25,26^ and the Onsager model.^27^ [It should be noted that, historically, both models were related to systems of electric dipoles. The magnetic analog of the Debye theory is the Curie–Weiss theory.]

In the Debye model, the effective dipole field is expressed in the form of the Lorentz equation, *H*_eff_ = *H* + (4π/3)*M*. The permeability in the Debye model reads

3 $$\mu_{D}=\frac{1+2y}{1-y}$$

This classical result has a well-known problem associated with the phase transition to a spontaneously magnetized/polarized state that occurs at the Curie point when the denominator of eq 3 equals to zero. This state has never been observed in polar or magnetic fluids. In modern concentrated magnetic fluids, the parameter *y* can reach values of about 2–4, i.e., notably greater than unity.

In the Onsager model, the orientation of each dipole is determined by the combined action of the external field, ***H***, and the reaction field, ***R***, which is initiated when the dipole polarizes the surrounding polar medium.^27^ In the Onsager model, the permeability, μ_*O*_, satisfies the equation^27,28^

4 $$\left(\mu_{O}-1\right)\left(2\mu_{O}+1\right)/\left(9\mu_{O}\right)=y$$

In the theory of dipolar hard sphere liquids, there is another analytical model: the mean spherical model (MSM). The model based on a special closure of the Ornstein–Zernike equation was solved by Wertheim in his seminal work.^29^ The expression found for the permeability is also determined by the single parameter *y*

5 $$\mu_{MSM}=q\left(2\xi \right)/q\left(-\xi \right),\;q\left(2\xi \right)-q\left(-\xi \right)=3y,\;q\left(x\right)=\frac{{\left(1+2x\right)}^{2}}{{\left(1-x\right)}^{4}}$$

Pshenichnikov compared the predictions of three analytical models with experimental data for moderately concentrated ferrofluids.^7^ The initial susceptibility was studied over wide ranges of the magnetic phase volume fraction [0.001,0.18] and temperature [260,380] K. For the most concentrated samples, the Debye parameter reached values of *y* ∼ 2. Contrary to the predictions of the Debye model, the permeability μ remains finite at any values of concentration. The Onsager model significantly underestimates the susceptibility values compared to the experimental data. This behavior is particularly evident in the dependence of permeability on concentration. According to eq 4, as concentration increases, so does permeability, and the latter approaches asymptotically to (9/2)*y*, meaning a linear dependence on concentration, μ*_O_* ∼ *y*. In real ferrocolloids, however, this dependence is only observed for diluted samples with *y* ≤ 0.1. Numerous experiments^7,8,30^ indicate that the permeability of moderately and highly concentrated MF is *quadratically* dependent on concentration, μ ∼ *y* ^2^, rather than linearly dependent.

Pshenichnikov et al. first observed this property in refs 7 and 13. To explain it, they proposed writing the effective field acting on magnetic moment in the modified form as *H*_eff_ = *H* + (4π/3)*M*_*L*_, where *M*_*L*_ = χ_*L*_ *H* is the Langevin magnetization in the system of noninteracting dipoles. In this way, for the permeability they obtained the result of the first-order modified mean field theory (MMFT1)

6 $$\mu_{P}=1+3y\left(1+y\right)$$

which described the experimental data particularly well.^13^ The formula [Disp-formula eq6] can be considered a two-term virial expansion of μ on powers of *y*, which is common for all three mentioned models: the Debye and Onsager models and the MSM.

Later, Ivanov and Kuznetsova^5^ developed perturbatively the second-order modified mean field theory (MMFT2) and corrected the result [Disp-formula eq6]:

$$\mu_{\mathrm{uni}}=1+3y\left(1+y+\frac{1}{16}y^{2}\right)$$ 7

The additional term in eq 7 compared to eq 6 is the contribution related to the third virial coefficient. This term was first obtained by Jepsen in ref 31. We note that in the three lowest orders the virial expansion of the mean-spherical model result [Disp-formula eq5] reproduces eq 7. The index at μ indicates the *universal* behavior of permeability, independent of short-range effects.

Until now, we have completely ignored the short-range correlations associated with the coupling parameter λ. For a dipole-hard sphere fluid, these correlations were considered in ref 32 in the form of a general virial expansion. Let us consider this issue in more detail.

The general form of the virial expansion of μ up to $O\left(y^{3}\right)$ reads^32^

8 $$\mu_{\mathrm{vir}}=1+3y+3y^{2}\left(1+b\right)+3y^{3}\left(\frac{1}{16}+2b+b^{2}+c\right)+...$$

where *b* is the second virial coefficient and *c* is the λ-dependent part of the third virial coefficient related to the irreducible three-vertex diagram (see details in the Supporting Information). We remind the reader that the terms “second” and “third” virial coefficients are not defined by eq 8 for permeability μ, but rather by the Debye-type relation for (μ – 1)/(μ + 2). To avoid confusing terminology, we determine new virial coefficients according to equation

9 $$\mu_{\mathrm{vir}}=1+3y\left(1+By+Cy^{2}\right)+...$$

where the introduced second and third virial coefficients, *B* and *C*, are related to the original ones by the obvious relations:

10 $$B=1+b,\;C=\frac{1}{16}+2b+b^{2}+c$$

The coefficient *b* was first calculated analytically by Buckingham and Joslin in ref 33 and later by Huke and Lücke^34^ as a series expansion in powers of λ:

11 $$b=\frac{\lambda^{2}}{25}+\frac{4\lambda^{4}}{1225}+\frac{\lambda^{6}}{6615}+\frac{37\lambda^{8}}{8004150}+O\left(\lambda^{10}\right)...$$

The coefficient *c* was found in ref 32 up to $O\left(\lambda^{11}\right)$

12 c=−0.09638896λ+0.00017068λ2−0.01626308λ3−0.002651575λ4−0.00059264489λ5−0.00050642297λ6+0.00009809470λ7−0.00005803783λ8+0.00001696677λ9−0.00000491826λ10+0.00000149673λ11+O(λ12).

The behavior of the virial coefficients (eq 10) is shown in Figure 1 as a function of the coupling parameter.

**Figure 1:**
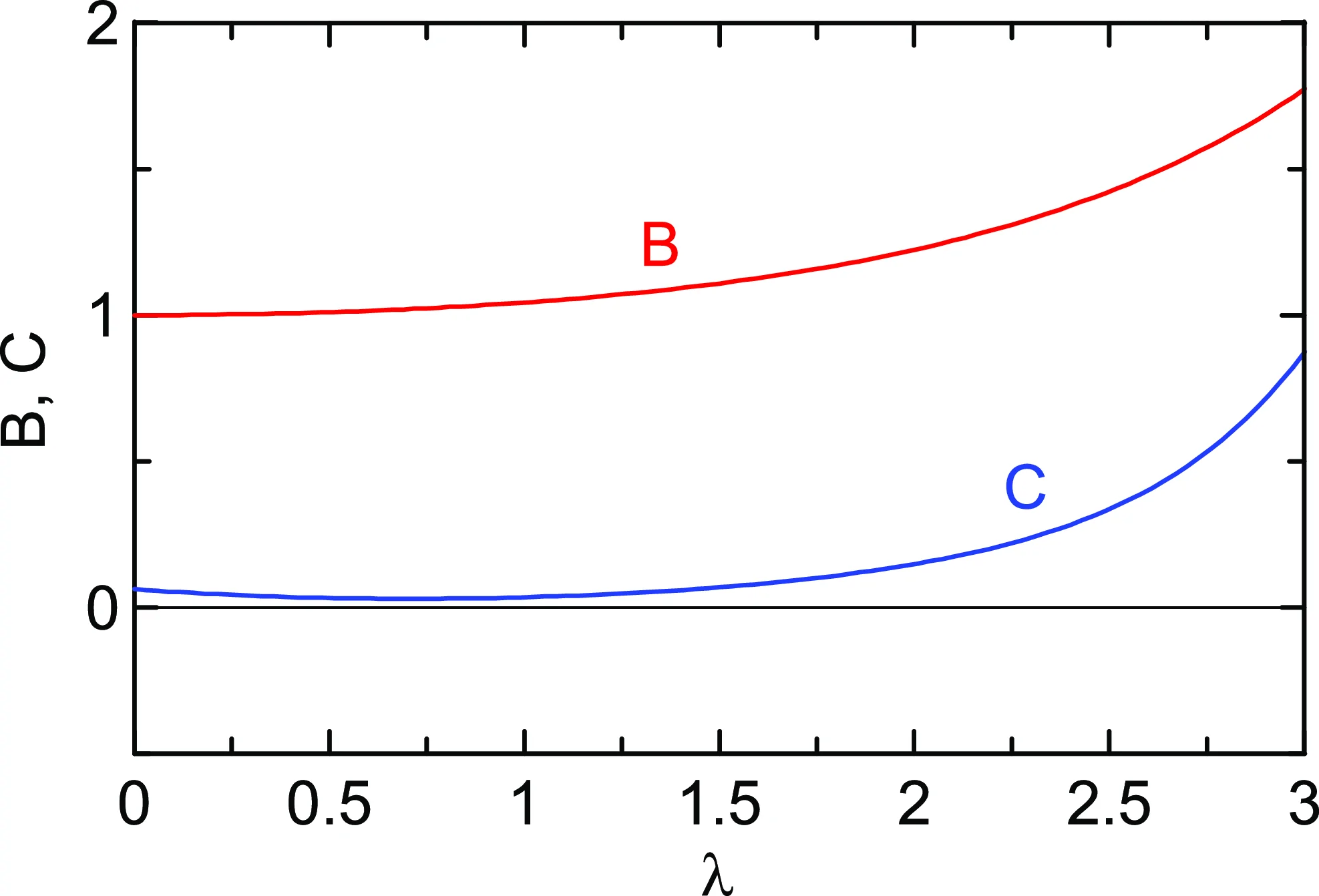
Second and third virial coefficients of expansion [Disp-formula eq9] as a function of the coupling parameter.

Note that for values of *y* ∈ [0,3], the third virial coefficient *C* decreases from its value of 1/16 at λ = 0, passes through a minimal value of 0.03 at λ = 0.79, and then increases. The growth of both coefficients becomes exponential for λ > 1 owing to the formation of chain structures^22^ with head-to-tail orientation of the magnetic moments, which minimizes the magnetic energy of a cluster of two or three particles. Comparing eqs 9 and 7 one concludes that virial expansion reduces to the MMFT2 when the short-range effects are completely neglected.

Strictly speaking, the applicability of virial expansions, such as those in eqs 7 or 9, is determined by the smallness of the parameters *y*, *yB,* and *yC*. However, due to the impossibility of examining the subsequent terms in the virial series, these relations are employed in practice even when the parameters *y*, *yB*, *yC* ≥ 1. A detailed comparison^20^ of numerical and experimental data with the predictions of the Langevin (eq 1), Debye (eq 3), mean-spherical (eq 5) models and expansions [Disp-formula eq7] or 9 revealed that the universal relation (eq 7) is an excellent approximation for ferrofluid permeability when *y* ≤ 2. For higher values of the parameter *y*, the expression [Disp-formula eq7] provides a reasonable lower bound estimate of μ. To more accurately describe μ, Ivanov and Kuznetsova^5^ added a term associated with the second virial coefficient, i.e., the short-range effects:

13 $$\mu_{\mathrm{IK}}=1+3y\left[1+y\left(1+b\right)+\frac{1}{16}y^{2}\right]$$

They found good agreement between eq 13 and the MC data of ref 15 for a dipolar HS fluid with the parameters *nd* ^3^ = 0.8 and *y* ≤ 2.5. Later, Lebedev studied the initial susceptibility of concentrated MF in a wide range of temperatures.^30^ In the experiments, he used magnetic fluids based on isooctane, which has a very low freezing point, *T*_*f*_ = −107°C. At low temperatures ∼−50°C, the magnetic permeability and parameter *y* of the most concentrated sample reached record values μ  ≈  150 and *y*  ≈  4.5, respectively. The author used the relation ([Disp-formula eq13]) to describe the observations and also obtained the satisfactory results for the permeability μ.^30^

Let us comment on the aforementioned results and emphasize the differences in existing representations of the permeability μ. The virial expansions [Disp-formula eq8] or 9 that take into account terms up to third order in concentration *n* ∼ *y* are formally *exact* relations. Using the diagrammatic representation^32^—see the Supporting Information—these decompositions account for *all* diagrams with two and three vertices. If, in the virial expansions, we selectively retain all two-vertex diagrams and the only three-vertex Jepsen diagram^31,32^ we obtain the result [Disp-formula eq13]. In turn, result [Disp-formula eq7] follows from eq 13 if one neglects all diagrams containing two or more dipolar (solid) lines between vertices. The better agreement between representations [Disp-formula eq7] and 13 and experimental and calculated data compared to the “exact” expansion [Disp-formula eq8] implies that the contributions of the diagrams they selectively consider are predominant. Therefore, one could argue a posteriori that the contributions of the discarded diagrams must somehow be leveled out in higher orders of the virial expansion.

There is another, hybrid version of the perturbation theory, an inherent part of which is the MC modeling. This is the Tani-Henderson-Barker-Hecht (THBH) theory developed in ref 35. The essence of the theory can also be understood using the virial expansion formalism [Disp-formula eq8]. Like the MMFT, the model disregards diagrams with multiple dipolar lines between vertices. The only difference between the two models relates to the third-order Jepsen diagram decorated with additional *g*_HS_(*r*_*ij*_) factors between nodes (see the Supporting Information (SI)). Here *g*_HS_(*r*) is the radial distribution function of a system of *hard spheres* with no dipoles present.^35^ In other words, the THBH model takes short-range correlations into account differently than the virial expansion does—not through the λ-parameter, which characterizes the dipole interaction in contact, but rather through the concentration n_*_ = *nd* ^*3*^, which controls the packing of hard spheres. The radial distribution function was determined in ref 35 in MC calculations. The final result for the permeability is

14 $$\mu_{\mathrm{THBH}}=1+3y\left(1+y+py^{2}\right)$$

where the *third virial coefficient* *p* is related to the three-particle perturbation integral *I*_*ddΔ*_ tabulated in ref 35. Also in^35^ the Padé approximation for *p* was found:

15 $$p=\frac{17}{16}\frac{1-0.93952n_{*}+0.36714n_{*}^{2}}{1-0.92398n_{*}+0.23323n_{*}^{2}}-1$$

We note that the parameter *p* is specific to eqs 14 and 15 and should not be confused with pressure. According to eq 15, it is a monotonically increasing function of n_*_. In the dilute limit, n_*_ → 0, the coefficient *p* equals to its Jepsen value of 1/16 and eq 15 reduces to eq 7. Thus, when short-range correlations are neglected, the models under consideration yield a universal dependence μ = μ_uni_(*y*). This dependence becomes two-parametric, i.e., μ = μ (*y*, λ), or, equivalently, μ = μ­(*y*, *n*), when short-range correlations become significant. The condition under which this occurs is formulated in Section 3 below. Additionally, we took into account that, of the three parameters *y*, n_*_, and λ, only two are independent, as *y* = 4πn_*_λ/9.

Interestingly, the predictions of eq 14 are in good agreement with the data from the aforementioned work^15^ as are the results [Disp-formula eq13] of Ivanov and Kuznetsova. Indeed, when we substitute the data for the most polar sample studied, n_*_ = 0.8 and *y* = 2.5, we find μ_IK_ = 35.8 and μ_THBH_ = 39.1. The difference in the predictions is less than error bar (±4), as indicated in ref 15. Despite the close similarity of the two models’ results, they describe *fundamentally different* behavior of permeability in highly polar samples. To discover this, consider samples with the *same* values of the parameter *y* and *different* values of the parameter λ. Consider two different values of the coupling parameter, λ_1_ > λ_2_, taken at some fixed value of *y*. According to expression [Disp-formula eq13], we have *μ*_IK_(*λ*_1_) > *μ*_IK_(*λ*_2_). However, the predictions of eq 14 are *opposite* to this. Indeed, the condition λ_1_>λ_2_ taken at fixed value of *y* means that the corresponding concentrations are related as *n*_1_<*n*_2_. The third virial coefficients in eq 14 then satisfy the inequality *p*(*n*_1_) < *p*(*n*_2_). Finally, we get: *μ*_THBH_(*λ*_1_) < *μ*_THBH_(*λ*_2_). Thus, to determine the superior approach, one must have extensive and accurate data on highly polar systems across a broad spectrum of particle concentrations and coupling parameters. Fortunately, such data now exists.^21^ We will analyze it in the next section.

### Path to Real Polar Liquids

The results in ref 21 are the most detailed and accurate set of MC calculations for the permittivity of a dipolar hard sphere fluid. The results cover a wide range of fluid concentration, n_*_ ∈ [0,1], and coupling parameter, λ ∈ [0,4]. Notably, within the experimental error, the data coincide with some previously known data. All the MC data are depicted in Figure 2 by symbols. The color of each symbol corresponds to a specific coupling parameter value. The black line denotes the universal dependence [Disp-formula eq7]. The error bar is only shown for cases with *y* ≥ 2.5, as this is where it becomes notable due to MC sampling difficulties at high values of *y* and n_*_.

**Figure 2:**
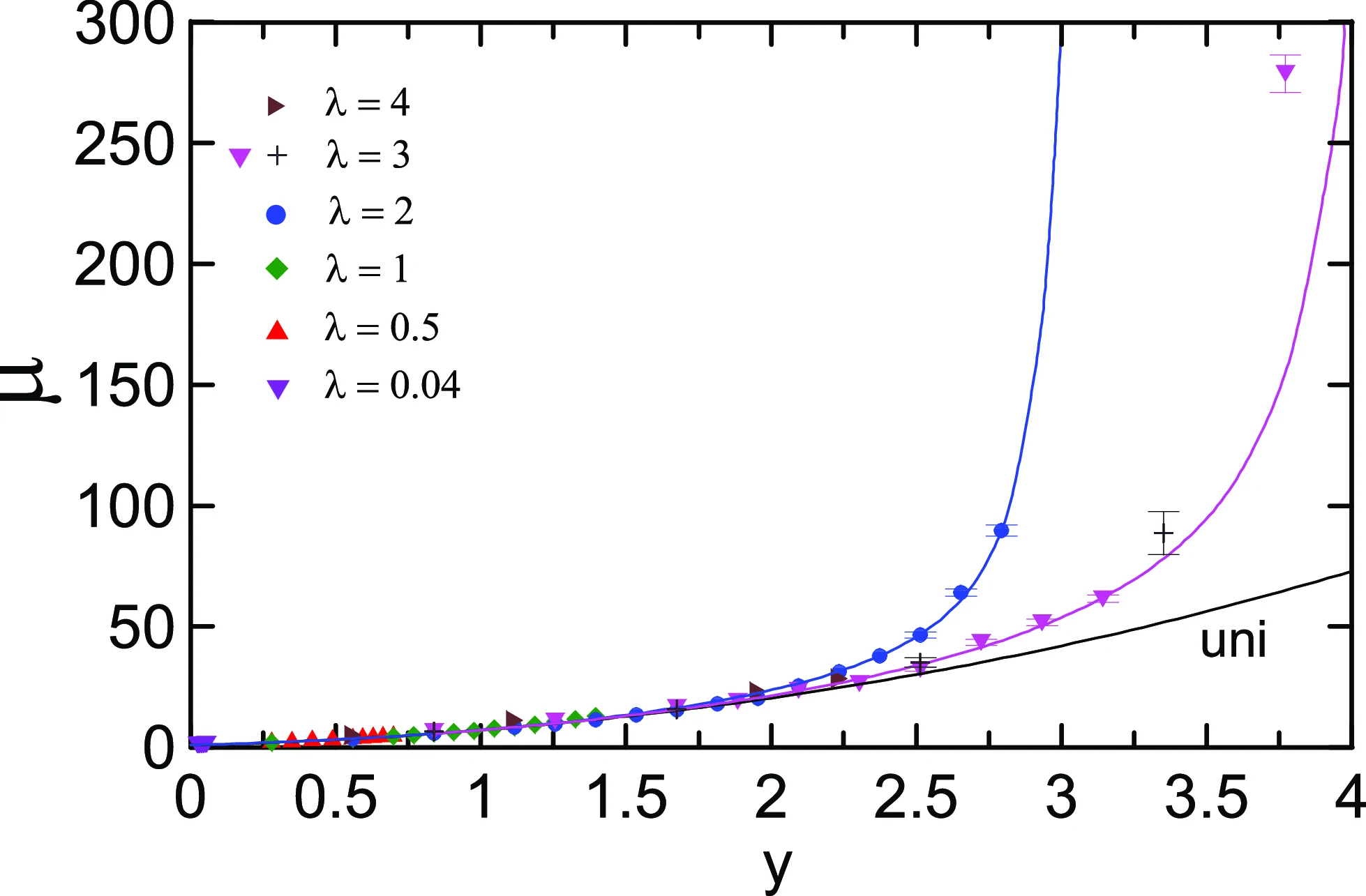
Permeability of the dipolar hard spheres versus the parameter *y* for several values of the coupling parameter λ, as indicated in the inset. The MC data are marked by symbols. The main data are taken from.^21^ The data marked by pluses are taken from ref 19 and are shown for comparison. The theoretical curves are plotted by solid lines. The black line corresponds to the universal dependence (eq 7). The colored lines satisfy eq 16. The blue and magenta curves are plotted for λ = 2 and 3, respectively. The error bar is only given for cases with *y* ≥ 2.5. In other cases, the error bar is significantly smaller than the symbol size.

Several characteristic features of the behavior of the permittivity can be drawn. (i) For *y* ∈ [0,2] the MC data is nearly independent of the coupling parameter, λ, and can be well approximated by the universal dependence (eq 7). (ii) For *y*  > 2 and λ  > 2, there is an explosive increase in permeability as *y* increases. It is important to note that *higher* values of λ correspond to *lower* values of permittivity.

The first observation plays a crucial role in our next consideration. Indeed, it determines the rough boundary of the applicability of the universal dependence (eq 7). When *y* ≤ 2, short-range effects associated with the shape of the molecules, the formation of clusters, or the off-center location of the dipole moment within the molecule are not important. Therefore, one should expect the relation [Disp-formula eq7] to apply to real polar liquids if this condition is met.

The second observation describes how quickly the deviation from the universal dependence occurs at *y*  >  2. This observation aligns with the predictions of the THBH model. However, the power-law growth of eq 14 is too slow to describe the data. A better agreement with simulations is achieved by raising the last term in eq 14 to an exponent:

16 $$\mu =1+3y\left(1+y\right)+\frac{6}{p^{2}}\left[\exp\left(p^{3}y^{3}/2\right)-1\right]$$

A similar procedure was used earlier in ref 18 to fit the MC data, although a different coefficient was used in the exponent. The blue and magenta solid lines were plotted in Figure 2 according to (eq 16) for the coupling parameters λ = 2 and λ = 3, respectively. Both curves fit the MC data well. However, for the maximum values of n_*_ = 1 and λ = 3, the parametric dependence (eq 16) somewhat underestimates the result for permittivity.

Using the fitting relation [Disp-formula eq16], it is possible to gain a qualitative understanding of the emergence of the criterion *y* ≤ 2 for the applicability of the universal relation [Disp-formula eq7]. Indeed, the rapid growth of permittivity begins when the exponent is of order of unity, i.e., at *py* ∼ 1. According to eq 15, at high concentration, n_*_ ≈ 1, the THBH third virial coefficient *p* ≈ 1/2. In this way, we obtain the mentioned criterion.

To examine the data in more detail, the following figures use a logarithmic scale for the permeability values. Figure 3a and 3b show the MC data from refs 19 and 21 for the coupling parameter λ = 2 and λ = 3, respectively, represented by the same symbols as in Figure 2. The approximation (eq 16) is shown by a solid line of the corresponding color: blue in a) and magenta in b). The color dotted lines are the result of the Tani-Barker-Henderson-Hecht theory (eq 14). The red lines correspond to the predictions of the Ivanov-Kuznetsova theory^5^ (eq 13). The universal dependence (eq 7) is depicted by the solid black line.

**Figure 3:**
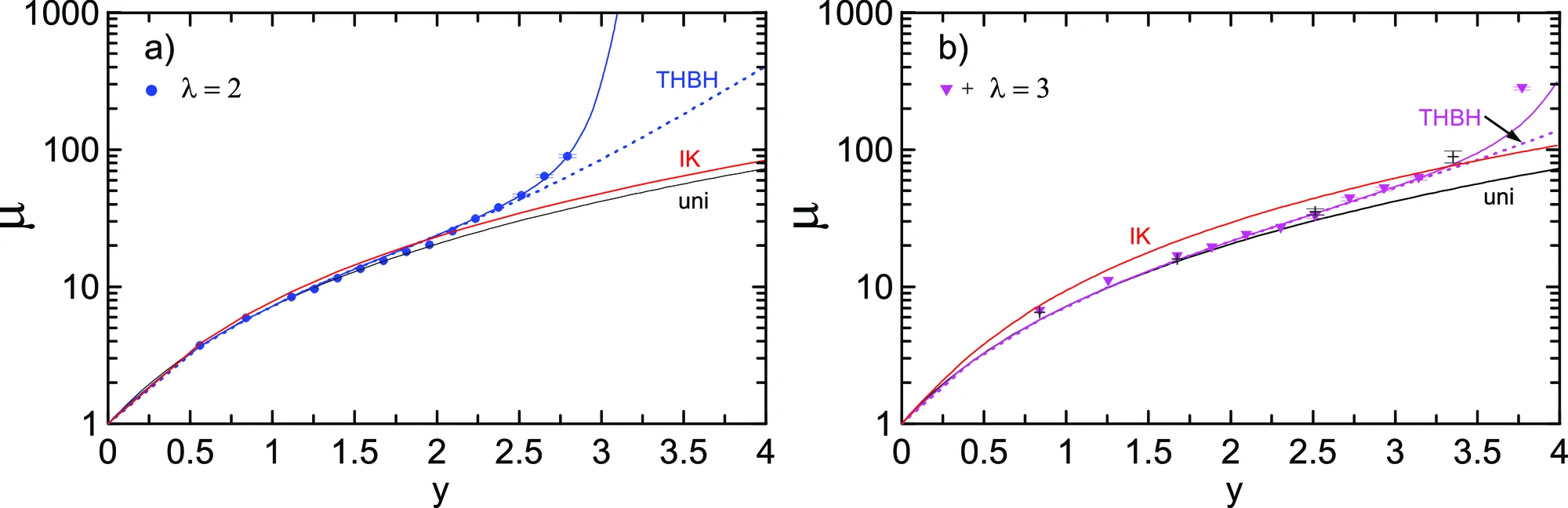
Permeability of the dipolar hard spheres versus the parameter *y* for several values of the coupling parameter λ, as indicated in the inset: a) λ = 2; b) λ = 3. The MC data are marked by symbols as in Figure 2 and are taken from refs 19 and 21. The dependencies (eq 16) are shown as blue and magenta solid lines in a) and b), respectively. The dotted lines show the predictions of the Tani-Barker-Henderson-Hecht theory (eq 14). The red lines correspond to the predictions of the Ivanov-Kuznetsova theory^5^ (eq 13). The black line is the universal dependence (eq 7).

As can be seen from the figures, dependence [Disp-formula eq16] perfectly approximates the numerical data for almost all values of the Debye parameter *y* ∈ [0,4]. Clearly, the THBH theory agrees with the data for *y* ≤ 2 but underestimates them for *y*  > 2. Meanwhile, the Ivanov and Kuznetsova model’s predictions are significantly worse. At λ = 2, the model slightly overestimates the calculations for *y*  < 2 and notably underestimates them for *y*  > 2. At λ = 3, the Ivanov and Kuznetsova theory greatly overestimates the calculation results at *y*  < 3.4, but underestimates them noticeably at *y* > 3.4. At the same time, the universal relation [Disp-formula eq7], regardless of the coupling parameter λ, approximates the data equally well when *y* ≤ 2.

The remainder of the present study is devoted to verifying the applicability of the universal dependence (eq 7) in describing the permittivity of real polar liquids. First, we determine the effective value *y*_eff_ of the Debye parameter, which considers the polarizability of real molecules, and specify which values of the dipole moments of molecules need to be used in calculations. Then, we compare the predictions of the universal dependence (eq 7) with the experimental data for more than hundred polar liquids and establish generally a good agreement between them. Finally, we summarize our results and draw conclusions.

## Results and Discussion

To describe the dielectric constant of polar liquids, we will transition from magnetic quantities to electrical ones. Instead of magnetic permeability, we will now deal with the dielectric constant, *μ* → *ε*. In addition to having a permanent dipole moment (*m*), molecules of polar liquids also have polarizability (α). We use the same letter to designate the dipole electric moment as we did earlier for the MF to emphasize the continuity of the two problems—for electric and magnetic dipoles. An appearance of the molecular polarizability effectively changes the value of the dipole moment of molecules. We take this into account according to the Debye theory^25^ introducing instead the parameter *y* it effective value:

17 $$y_{\mathrm{eff}}=4\pi n\alpha /3+4\pi nm^{2}/\left(9k_{B}T\right)=y_{\alpha }+y_{m}$$

Here, we determined the Debye parameters separately: *y*_*α*_ = 4*πnα*/3 and *y*_*m*_ = 4*πnm* ^2^/(9*k*_*B*_ *T*); these parameters are related to the two mechanisms by which the dipole moment of polar liquid molecules appears. According to the Debye theory, the polarization contribution *y*_α_ is determined by the Clausius–Mossotti relation^28,36^

18 $$y_{\alpha }=\frac{\varepsilon_{\infty }-1}{\varepsilon_{\infty }+2}$$

where *ε*_∞_ is the high-frequency dielectric constant, which is usually approximated by the squared optical refractive index.^28^ However, to make both mechanisms equivalent when describing permittivity, we proceed differently here. Below, we define *y*_α_ according to the same relation ([Disp-formula eq7]), taken in the high-frequency limit, when the mechanism associated with the reorientation of permanent dipoles becomes inactive:

19 $$\varepsilon_{\infty }=1+3y_{\alpha }\left(1+y_{\alpha }+\frac{1}{16}y_{\alpha }^{2}\right)$$

It should be noted that using eq 19 instead of [Disp-formula eq18] leads to a slight increase in the estimate for *y*_α_. According to the data for 101 liquids given in Table S1 of the Supporting Information, *ε*_∞_ ∈ [1.8, 2.4]. The Clausius–Mossotti relation (eq 18) gives *y*_*α*_ ∈ [0.21, 0.32] whereas our relation ([Disp-formula eq19])— *y_α_* ∈ [0.22, 0.35]. Apparently, this difference of just 4–8% is difficult to detect experimentally, particularly in comparison with the more noticeable dipole contribution *y*_*m*_.

Finally, the universal dependence (eq 7) for the permittivity of the polarizable polar liquids takes a form:

20 $$\varepsilon_{\mathrm{uni}}=1+3y_{\mathrm{eff}}\left(1+y_{\mathrm{eff}}+\frac{1}{16}y_{\mathrm{eff}}^{2}\right),{\;y}_{\mathrm{eff}}=y_{\alpha }+y_{m}$$

The most complex issue in applying eq 20 to real systems is the dipole moment of molecules. Unlike magnetic fluids, the dipole moment of polar liquids is not uniquely determined. In polar liquids, the dipole moment of the molecules generally depends on neighboring molecules. Only in the rarefied gas phase, when intermolecular distances are large, does the dipole moment of isolated molecules take on a very definite value, becoming an unambiguously determined quantity, *m*_*g*_. The dipole moment of a molecule can also be determined in the condensed phase. One of the main methods for measuring the dipole moment *m*_*l*_ in liquids was proposed by Debye.^37^ This method involves measuring the polarization in an external field of dilute mixtures of polar liquids in nonpolar ones, such as benzene, tetrachloromethane, and some others. It was assumed that such solutions would mimic the situation typical of gases. Debye himself noted that, in some cases, the dipole moments found in gases and solutions differ, *m*_*l*_ *≠ m*_*g*_. However, the difference was small and attributed to insufficient measurement accuracy.^37^ Subsequent experimental studies established the existence of this difference, leading to the terms “true” and “apparent” dipole moments for *m*_*g*_ and *m*_*l*_, respectively, as defined in.^38,39^ By the beginning of the 1960s, the physical origin of the mismatch had already been established. So, MaClellan noted that “··· the dipole moment values are subject to solvent effect which depends on interaction of the test substance with the solvent”.^40^ Since then, the term “dipole moment in solution” has become widely used.

The values of the dipole moments of some polar liquid molecules in the gas phase and in a benzene solution are given in Table 1. As the table shows, dipole moments of molecules in the condensed phase can be greater or less than their gas-phase values. Due to the strong dependence ([Disp-formula eq20]) on the dipole moment, the difference Δ*m* = *m_l_* – *m*_*g*_ = ±(0.1–0.2) D can be notable when calculating the permittivity of polar liquids. In early studies, the change in dipole moment in solution was associated with the nonspherical shape of the solute molecules. Theoretically, this effect was considered within the Onsager model’s generalization for ellipsoidal molecules.^28,41^

**Table 1:** Dipole Moments of Some Polar Liquid Molecules in the Gas Phase, in a Benzene Solution and in Pure Liquid at *T* = 25 °C^a^

	*m*_*g*_	*m*_*l*_		*m*_*p*_	
substance	[D]	[D]	ref.	[D]	ref.
chlorobenzene	1.69	1.58	^40^		
cyclohexanone	3.25	3.08	^40^		
tetrahydrofuran	1.75	1.75	^40^		
isopropylamine	1.19	1.45	^43^		
ethanol	1.69	1.73	^40^	2.56	^44^
1-propanol	1.57	1.70	^40^	2.56	^44^

a The data for *m_g_* are taken from ref 42.

An important property of the dipole moments of some organic molecules is their relationship to the molecule’s spatial structure (stereoisomerism) of the molecule. This relationship was first established in 1932 by Mizushima and Higasi in their pioneering work.^45^ [Note that a recent review by Toyota^46^ includes Mizushima and Higashi’s article in its entirety.] The authors demonstrated that the dipole moments of 1,2-dichloroethane (DCE) depend on both temperature and solvent. So, in ref 47 they showed that the dipole moment of DCE in heptane increased from 1.16 to 1.46 D with the temperature growth from 223 °K to 323 °K. This effect is explained by the existence of conformers with different dipole moments, the relative population of which depends on the temperature and the solvent used. In ref 48 the dipole moments of 1,2-dichloro-, 1-chloro-2-bromo-, and 1,2-dibromopropane were determined in solutions of benzene and tetrachloromethane. The values of the dipole moments in benzene were found to be 0.3–0.4 D higher than in tetrachloromethane, due to a higher concentration of more polar conformers in C_6_H_6_ compared to CCl_4_.^48^

However, it should not be assumed that the dipole moments of molecules in solutions are generally poorly defined quantities or that there is always a significant difference in the values of the moments determined in different solutions. In fact, a noticeable difference exists for very few liquids, particularly the aforementioned stereoisomers. In most cases, the dipole moment values found in solutions of benzene, tetrachloromethane, *n*-hexane, etc., are in good agreement with each other.^40^ We also note that benzene is the most popular nonpolar solution for determining the dipole moment of molecules. Approximately 90% of the experimental data collected by McClellan^40^ were obtained in benzene, which can be considered the standard solution for determining the dipole moment of molecules.

Finally, we note that for some liquids there is a problem determining the dipole moment *m*_*p*_ of molecules of *pure* polar liquids, which may also differ from its value measured in a solution of nonpolar liquids. This property is typical of polar liquids with hydrogen bonding. A prime example of such a medium is water. In refs 49 and 50 Gubskaya and Kusalik numerically demonstrated that the dipole moment of a liquid water molecule is 2.9 D, which is 1.05 D greater than the value observed in the gas phase. The dielectric properties of water, however, are extreme and will not be discussed in this study. By contrast, we will be interested in alcohols below. The dipole moments of a few alcohols were studied in the pioneering numerical modeling^44^ by Jorge et al. It was shown that local polarization caused by the formation of hydrogen bonds leads to an increase in the dipole moment of molecules by approximately 0.9 D in comparison with their value in the gas phase—see the data for ethanol and 1-propanol in Table 1. Thus, the situation with the dipole moments of pure liquids is also not entirely clear.

To conclude the discussion of molecular dipole moment values, we emphasize once more that calculations according to eq 20 should use the pure liquid values of *m*. As these values are usually unknown, different strategies are employed for polar liquids with and without hydrogen bonding. (i) For polar liquids *without* hydrogen bonds, we use for *m* the values of the dipole moment determined in a *benzene* solution. (ii) For polar liquids *with* hydrogen bonds, the pure and solution-determined values of the dipole moment differ considerably as seen from Table 1. In this case, we have to rely on values of *m* obtained from a numerical experiment. The self-consistency of both assumptions is verified below. In particular, we demonstrate that the dipole moments used in (i) and (ii), and those calculated from the experimental permittivity values, are very close to each other, which confirms the strategies *a posteriori*.

Now, let us return to the main focus of the current study – the universal description (eq 20) of the permittivity of polar liquids. According to the Introduction, we expect it to be applicable for liquids with the effective Debye parameter *y*_eff_ ≤ 2. For the HS dipolar fluid, such values of *y*_eff_ correspond to the permittivity *ε* ≤ 30—see Figure 2. Thus, to verify eq 20, we selected the experimental data on the dielectric constant of approximately 100 polar liquids with *ε* ≤ 30.

Before analyzing the data and results, we would like to make an important remark. Much of the data on the permittivity of polar liquids was obtained nearly 100 years ago when the study of the properties of such liquids was particularly popular due to the work of Debye and Onsager. As we will see, only isolated data exist for a number of liquids, and these data have not been reexamined in the intervening years. Consequently, some data is significantly outdated and some is even erroneous (see, for example, the comment on Table S1 of the Supporting Information regarding the permittivity of 4-bromotoluene).

Similar to permittivity data, various sources exist for experimental values of the dipole moments of molecules in solutions. Our primary focus will be on the data collected by McClellan from the 1920s to the 1960s.^40^ Since these data are not always complete and unambiguous, we will also use several other sources, including original papers and data collected in ref 43. Among these papers, we will use the data obtained by Professor R. J. W. Le Févre (1905–1986) of the University of Sydney.^51–53^ He was involved in the experimental determination of molecular dipole moments for decades. In our opinion, he obtained the most detailed and accurate results for many families of polar liquids.

As an example, Table 2 presents data for 10 selected liquids. The full list of parameters for all 101 liquids is given in the Supporting Information.

**Table 2:** Summary of Liquid Parameters^a^

no	substance	*M* [g/mol]	ρ [g/cm^3^]	$\sqrt{\varepsilon_{\infty }}$	*m*_*l*_ [D]	ref.	*y*_eff_	*ε*_uni_	*ε*_exp_
1	bromoethane	108.965	1.4500	1.4204	1.93	^52^	1.28	10.1	9.01
2	bromobenzene	157.01	1.4886	1.5568	1.55	^40^	0.82	5.54	5.40
3	ethyl acetate	88.106	0.8944	1.3695	1.84	^54^	0.94	6.61	5.99
4	methyl ethyl ketone	72.106	0.7999	1.3760	2.76	^40^	1.97	19.9	18.3
5	methyl propyl ketone	86.132	0.8041	1.3869	2.77	^43^	1.71	15.8	15.3
6	tetrahydrofuran	72.106	0.8833	1.4050	1.75	^40^	1.02	7.42	7.43
7	2-heptanone	114.185	0.8068	1.4063	2.71	^55^	1.32	10.6	11.7
8	*m*-chloroaniline	127.572	1.2107	1.5908	2.40	^56^	1.49	12.7	12.3
9	ethanol	46.068	0.7849	1.3590	1.73	^40^	1.27	10.1	24.9
					2.56	^44^	2.51	30.4	24.9
10	1-propanol	60.095	0.7997	1.3837	1.70	^40^	1.03	7.48	20.5
					2.56	^44^	2.03	21.0	20.5

a The molar mass *M*, mass density *ρ*, refractive index $\sqrt{\varepsilon_{\infty }}$ , dipole moment in solution, and the source of this value are indicated. Also given are the effective Debye parameter *y*_eff_, predicted *ε*_uni_ and experimental *ε*_exp_ values of the permittivity. The data for *M, ρ*, $\sqrt{\varepsilon_{\infty }}$ and *ε*_exp_ are taken mainly from ref 42 at a temperature *T* = 25 °C. The theoretical values are calculated according to the universal dependence (eq 20). For alcohols, the second (lower) value of the dipole moment is given for the pure liquid.

The data for the liquid molar mass *M*, mass density ρ, refractive index $\sqrt{\varepsilon_{\infty }}$ and permittivity *ε*_exp_ are taken from.^42^ For alcohols, two values of the dipole moment are indicated: the upper value is found in a nonpolar solution and the lower value is obtained numerically for a pure liquid. Columns *y*_eff_ and *ε*_uni_ determine the calculated values of the effective Debye parameter and the universal permittivity ([Disp-formula eq20]). For the first eight liquids, the theoretical predictions are in good agreement with the experimental data. We note that in all these cases the parameter *y*_eff_ < 2. The situation with two alcohols—samples 9 and 10 in Table 2—looks different. Substituting the dipole moments of the molecules determined in solutions—1.73 D for ethanol and 1.70 D for 1-propanol—into eq 20 yields strongly underestimated values of the dielectric constant, differing from the experimental values by a factor of 2.5–2.8. This discrepancy is related to the difference in dipole moment values found in nonpolar liquid solutions and pure liquids. Using the Self-Consistent Electrostatic Embedding method, Jorge et al. numerically determined the dipole moments of molecules in pure liquids. For ethanol and 1-propanol they found *m*_*p*_ = 2.56 D. Substituting this result into eq 20 yields new estimates of 30.4 and 21.0, respectively. The former slightly exceeds the experimental data; this is attributed to the large value of the parameter *y*_eff, eth_ = 2.5 > 2. The latter is in perfect agreement with the experimental data.

A comparison of the predicted permittivity of 101 polar liquids according to eq 20 and the experimental data is given in Figure 4.

**Figure 4:**
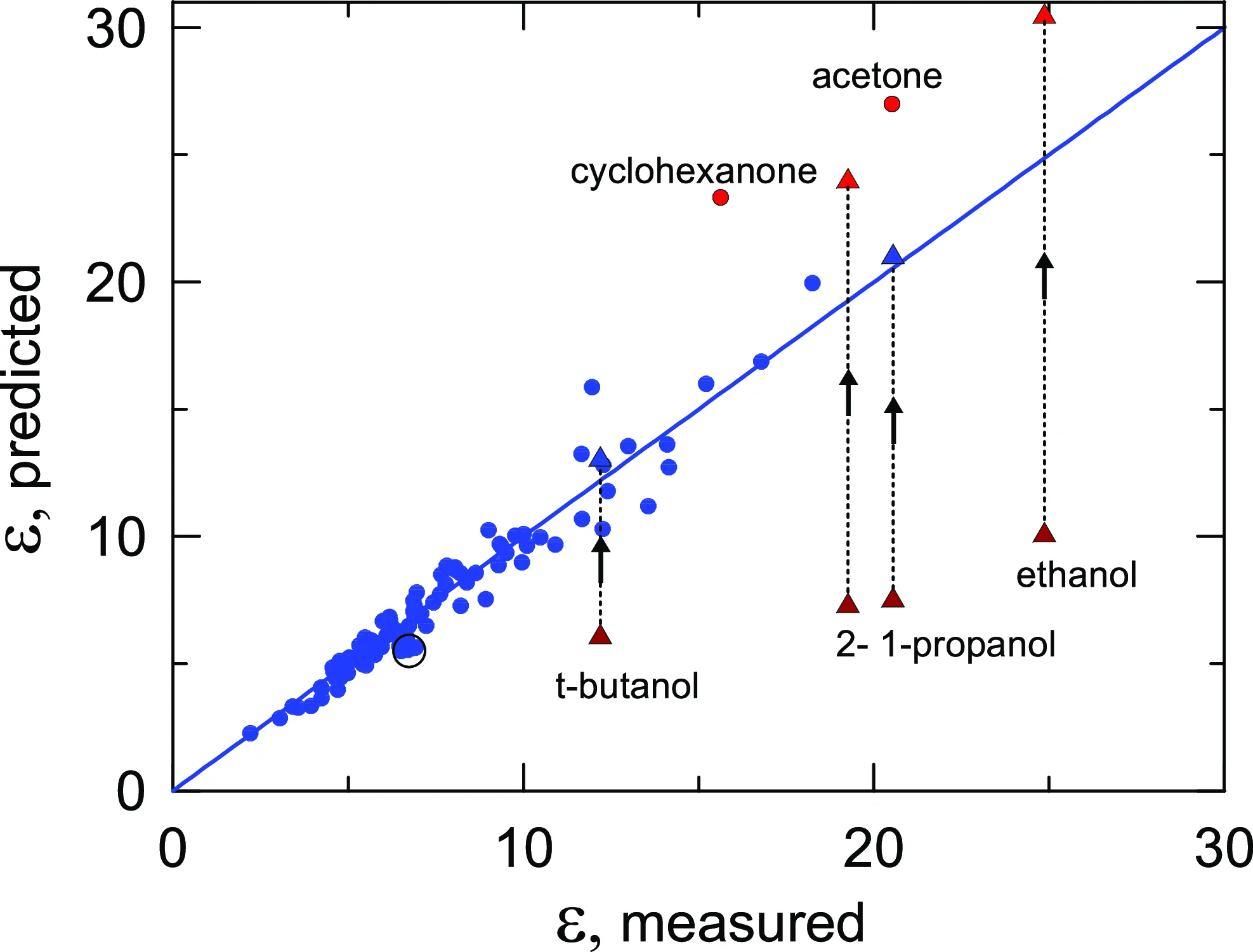
A comparison of the predicted permittivity of 101 polar liquids according to eq 20 and the experimental data. Data for aprotics and alcohols are shown by circles and triangles, respectively. The blue solid line corresponds to ideal agreement between the data. The blue and red symbols correspond to Debye parameter values less than and greater than two, respectively. The brown triangles mark the result found when the dipole moment of alcohols is determined in solution. Four arrows illustrate how this result changes when using values of the dipole moments for pure liquids. The empty black circle indicates six data points for 2-, 3-, and 4-bromo- and chloroheptanes, which were taken from ref 57.

Data for alcohols are shown by triangles, other data—by circles. The blue and red symbols correspond to Debye parameter values of less than 2 and greater than 2, respectively. The brown triangles mark the result found with the dipole moment of alcohols determined in solution. When the dipole moments of pure liquids^44^ are substituted into the eq 20, the predictions shift toward the blue solid line, as shown in Figure 4 by dashed lines with arrows. The predictions are notably higher than the line for ethanol with *y*_eff, eth_ = 2.5 and for 2-propanol with *y*_eff, pro_ = 2.2. Meanwhile, the Debye parameters for tert-butanol and 1-propanol are 1.5 and 2.0, respectively. Consequently, the predictions align well with the data.

Finally, we note in Figure 4 some peculiar deviations from the universal dependence ([Disp-formula eq20]). These deviations are marked by an empty black circle containing six data points for 2-, 3-, and 4-bromo- and chloroheptanes. The data on their permittivities and dipole moments were obtained nearly 100 years ago by Errera and Sherrill^57^ and have not been updated since. In our opinion, the dipole moment values indicated in this work are underestimated by approximately |Δ*m*| ≈ 0.15–0.2 D. Indirect evidence for this underestimation is provided by Errera and Sherrill’s other data found for 1-bromoheptane. They indicated a value *m*_*l*_ = 1.85 D for the dipole moment. A later study by Le Fevre and Williams^52^ gave a larger value of *m*_*l*_ = 1.97 D for the same substance. We use this result in our analysis.

Figure 5 depicts the comparison of the predicted and experimental values of the dipole moments of molecules. The theoretical values were calculated according to the universal dependence ([Disp-formula eq20]) and the experimental values of the permittivity indicated in the last column of Table 2. The full list of the parameters for all liquids is provided in the Supporting Information. The averaged experimental error in determining the dipole moment over 101 liquids is about Δ*m* = ±0.1 D.^40,43^ The two black dashed lines in Figure 5 indicate the Δ*m* = ±0.1 D interval around the idealized dependence for which the predictions match the data. As can be seen in the figure, only a very small amount of data falls outside the specified range. Apart from the aforementioned liquids with *y*_eff_ > 2, which are marked with red symbols, only 9 liquids have |Δ*m*| > 0.1 D. Notably, six of these liquids are the aforementioned 2-, 3-, and 4-bromo- and chloroheptanes, whose dipole moments are apparently underestimated.

**Figure 5:**
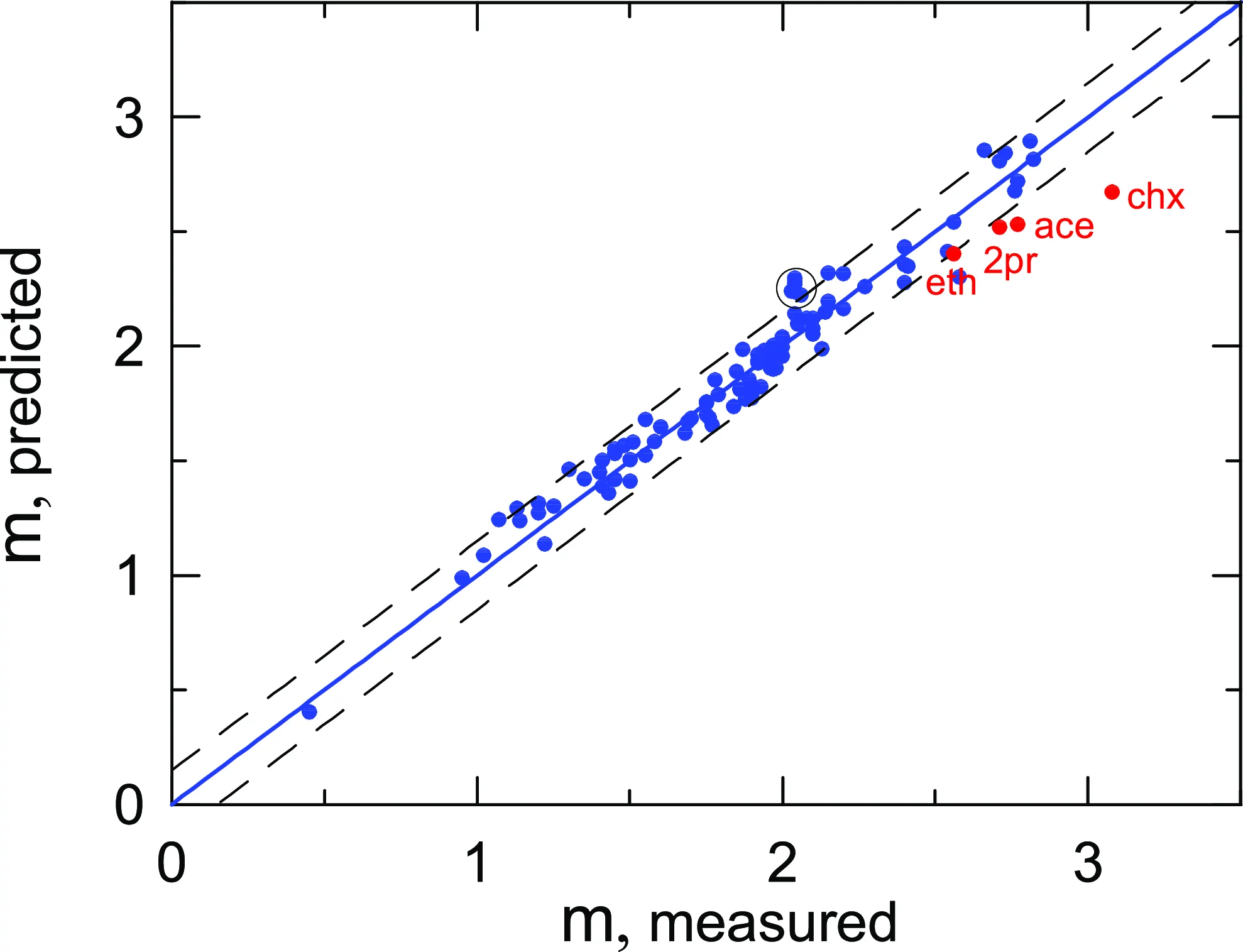
A comparison of the predicted dipole moments of 101 polar liquids according to eq 20 and the experimental data. The blue solid line corresponds to ideal agreement between the data. The black dotted lines are shifted from the blue one by the value Δ*m* = ±0.1 D. The blue and red symbols correspond to Debye parameter values less than and greater than 2, respectively. The data for four liquids are given explicitly. The notation used is eth for ethanol, 2pr for 2-propanol, ace for acetone and chx for cyclohexanone. The empty black circle marks six data points for 2-, 3- and 4- bromo- and chloroheptanes taken from ref 57.

It is also interesting to compare the results for the dipole moments of stereoisomeric liquids. For the latter, as mentioned above, we used experimental values found in benzene. For example, for 1,2-dichloropropane and 1,2-dibromopropane, the experimental values of *m*_*l*_ are 1.87 D^40^ and 1.43 D^48^ respectively. The values calculated according to the universal dependence ([Disp-formula eq20]) and the experimental values of the permittivity—see Table S1 in the SI—are 1.99 and 1.36 D, respectively, i.e., well agree with the experimental ones.

Until now, comparison of the universal dependence ([Disp-formula eq20]) with experimental data has been performed at a fixed temperature *T* = 25°C. However, the universal description remains accurate over a fairly wide temperature range. In Figure 6, the symbols indicate the experimental permittivities of three polar liquids—1-propanol, *m*-chloroaniline, and tetrahydrofuran—in the temperature range of [0,50] ^◦^C.

**Figure 6:**
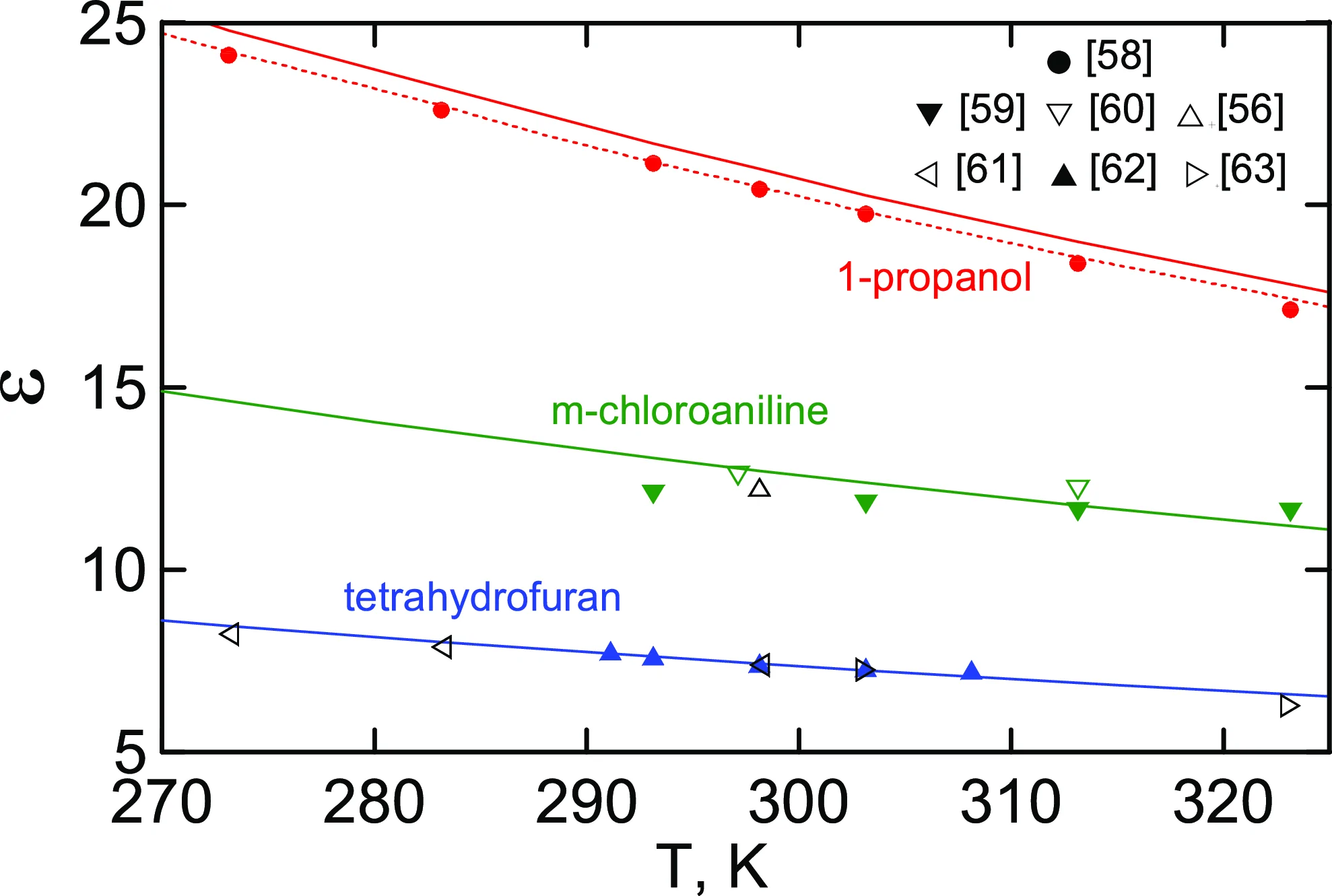
Permittivities of 1-propanol, *m*-chloroaniline, and tetrahydrofuran as a function of temperature. The inset shows the references of the data marked with symbols. The theoretical values (solid lines) are found according (eq 20) with the fixed dipole moments given in Table 2. The dotted line show the universal dependence (eq 20) found at lower value of dipole moment of 1-propanol *m* = 2.54 D instead of 2.56 D for solid line.^58,59,60,61,62,63^

The predictions are depicted by solid lines and found according eq 20. The calculations assumed a linear dependence of the molar volume and refractive index of liquids on temperature, while the dipole moment of molecules was considered constant and equal to the value specified in Table 2. As seen, the theoretical and experimental results are in a good agreement. Note that the predictions for 1-propanol are slightly higher than the measured values. However, if we use a 1% smaller value of dipole moment, *m* = 2.54 D instead of the value *m* = 2.56 given in Table 2, the results would agree almost perfectly as shown in Figure 6 by dotted line.

Summarizing the section, we once again note the good agreement between the universal relationship (eq 20) and the experimental data on the permittivity of many polar liquids. In the next section, we will consider a generalization of eq 20 to the case of mixtures of polar liquids.

### Permittivity of Liquid Mixtures

Due to the one-particle nature of the Debye parameters *y*_α_ and *y*_*m*_, which are related, respectively, to the polarizability and dipole moment of molecules, the universal relation (eq 20) allows a simple generalization to liquid mixtures:

$$\begin{array}{c} \varepsilon_{\mathrm{mix}}=1+3y_{\mathrm{eff},\mathrm{mix}}\left(1+y_{\mathrm{eff},\;\mathrm{mix}}+\frac{1}{16}y_{\mathrm{eff},\;\mathrm{mix}}^{2}\right),\\ y_{\;\mathrm{eff},\mathrm{mix}}=\underset{i}{\sum }y_{\;\mathrm{eff},i}=\underset{i}{\sum }\left(y_{\alpha ,i}+y_{m,i}\right) \end{array}$$ 21

Below we will test this relationship for binary mixtures, first a polar liquid in a nonpolar liquid, and then consider the case of both polar components. Let us denote the dielectric constants as *ε*_1_ and *ε*_2_, and the molar volumes of pure components as *V*_1_ and *V*_2_. The molar volume of mixture *V*_mix_ is considered additively:

22 $$V_{\mathrm{mix}}=xV_{1}+\left(1-x\right)V_{2}$$

where *x* is the mole fraction of the first component. Usage of eq 22 means disregarding the effect of a volume excess of mixture. In all the mixtures studied below—see Figures 7 and 8—the actual volume excess Δ*V*/*V* ≤ 0.003–0.008 and can be neglected.

**Figure 7:**
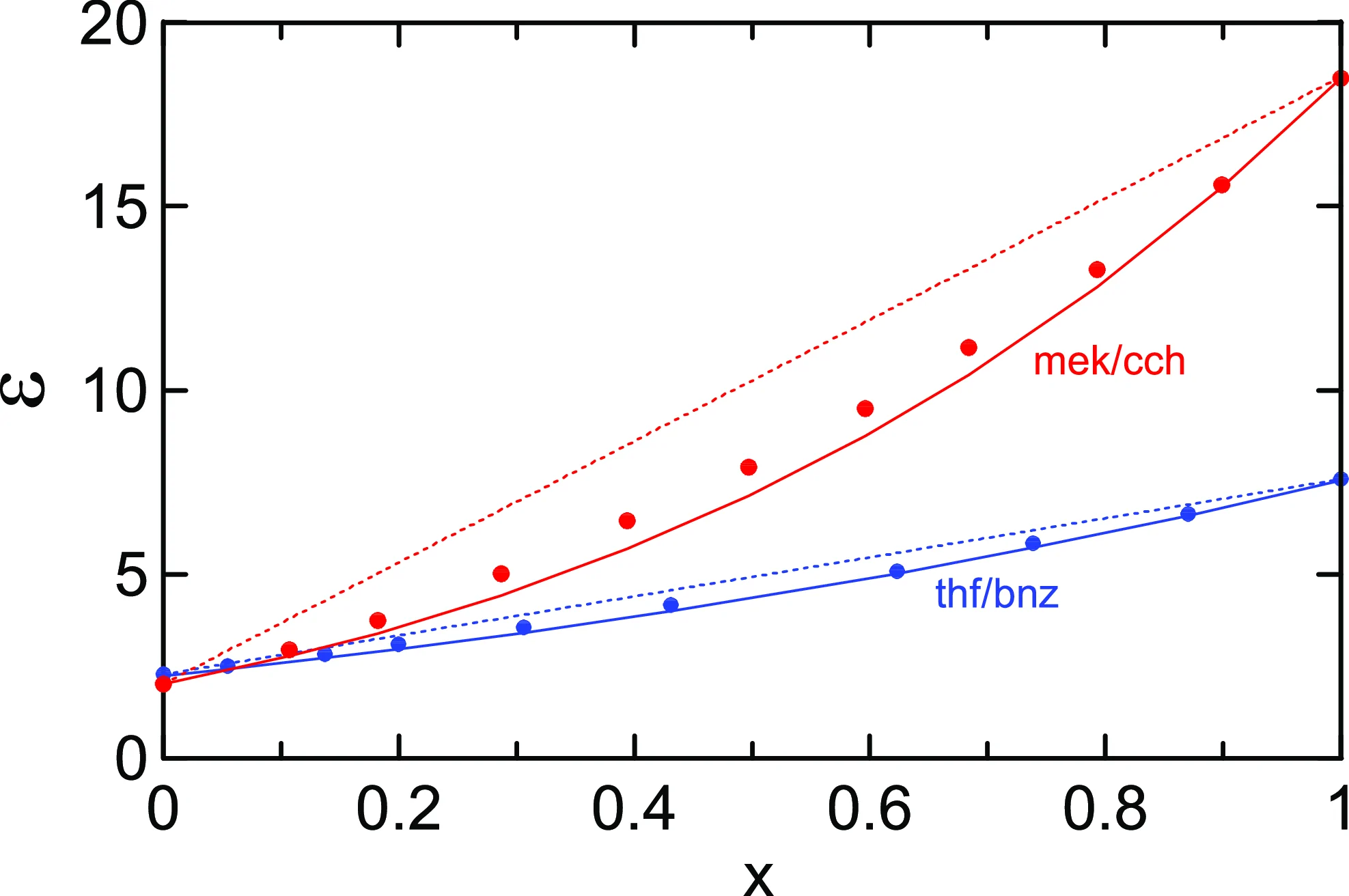
Permittivities of the mixtures of methyl ethyl ketone (mek) in cyclohexane (cch) and tetrahydrofuran (thf) in benzene (bnz) as a function of mole fraction of polar component. Symbols mark the experimental data of refs 64 and 65. The theoretical values are found according eq 21. The dotted lines show the average value *ε̅* of the permittivity.

**Figure 8:**
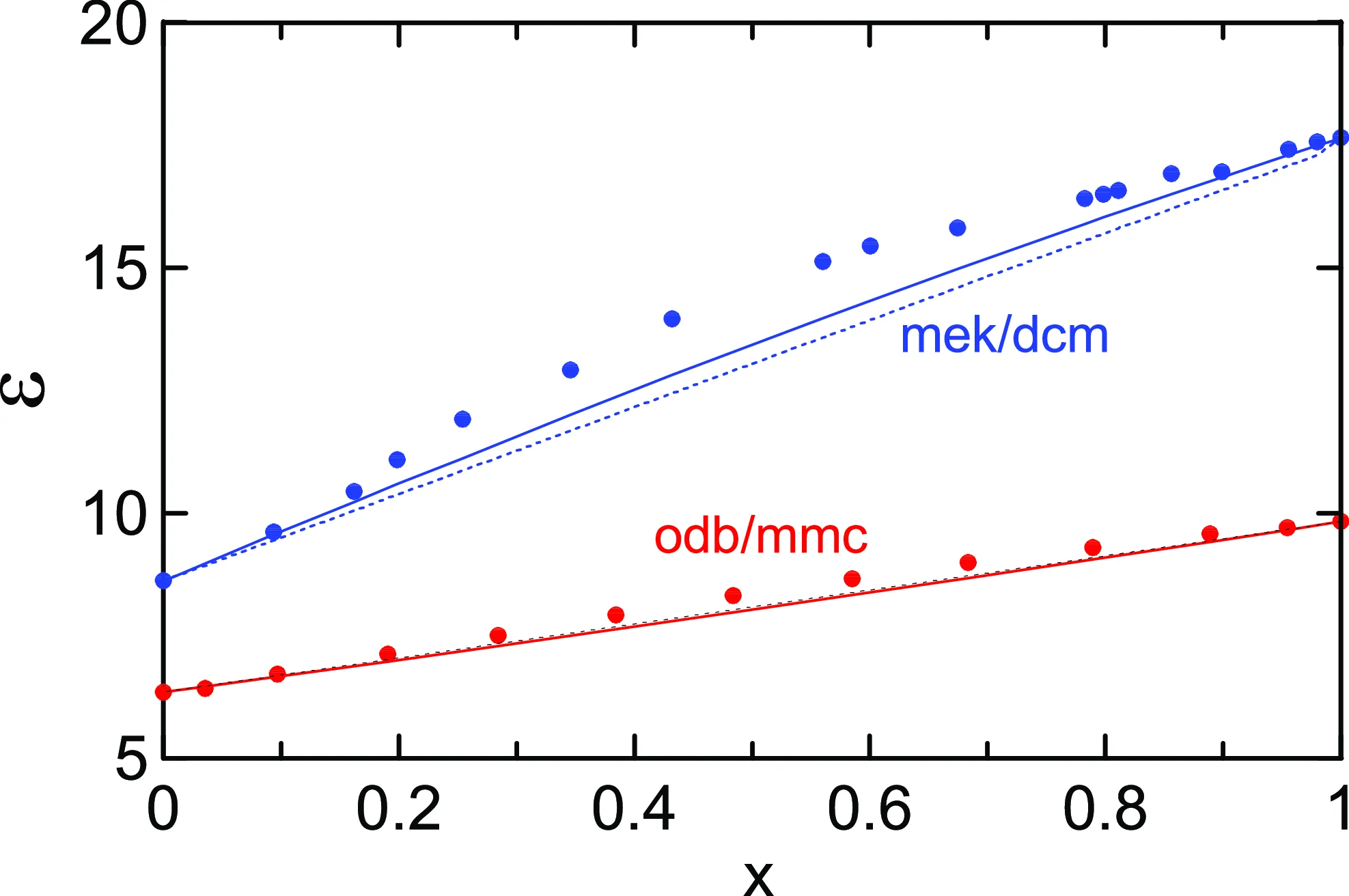
Permittivities of the mixtures of two polar liquids: methyl ethyl ketone (mek) in dichloromethane (dcm) and o-dichlorobenzene (odb) in methyl methacrylate (mmc) as a function of mole fraction of first component. Symbols mark the experimental data of refs 66 and 67. The theoretical values are found according eq 21. The dotted lines show the average value *ε̅* of the permittivity.

As polar components we will consider methyl ethyl ketone (MEK), tetrahydrofuran (THF), dichloromethane (DCM), o-dichlorobenzene (ODB), and methyl methacrylate (MMC). Here, we are interested only in the molar dependence of the permittivity of the mixture under the assumption that for pure substances it is ideally described by relations (eq 20). This means that for the dipole moments of molecules we use the following values: *m*_mek_ = 2.70 D, *m*_thf_ = 1.75 D, *m*_dcm_ = 1.68 D, *m*_odb_ = 2.26 D, and *m*_mmc_ = 1.85 D which are well correlated with the experimental values 2.76, 1.75, 1.55, 2.27, and 1.78 D, respectively, indicated in the Table S1 in the SI. The parameters of nonpolar components are found in.^42^ In Figure 7, circles show the experimental data for permittivity of methyl ethyl ketone in cyclohexane^64^ and tetrahydrofuran in benzene^65^ as a function of mole fraction of first (polar) component. The solid lines are plotted according to eq 21. The dotted lines show value of the permittivity averaged over the mole fractions of two components, *ε̅* = *xε*_1_ + (1 – *x*)*ε*_2_.

As Figure 7 shows, the theoretical and experimental data are in good agreement with each other. There is a slight excess of experimental values over predicted values for mole fractions 0.2 < *x* < 0.8. This excess disappears at *x*  <  0.2 and *x*  > 0.8. This behavior is due to the actual correlated arrangement of the mixture molecules. This property is not considered in relation [Disp-formula eq21], which assumes a *random* arrangement of the molecules in the mixture. For this reason, the calculated permittivity values approximate the experimental values from *below*, which appears to be a general property of mixtures.

In Figure 8, it is depicted the behavior of permittivity of mixture of two polar liquids. The data are taken from ref 66 for mixture of methyl ethyl ketone (MEK) in dichloromethane (DCM) and from ref 67 for mixture o-dichlorobenzene (ODB) in methyl methacrylate (MMA).

Similar behavior is observed again, as in the previous picture: the predictions are slightly below the data. The maximum deviation occurs at mole fractions *x* of about 0.5. At the same time, unlike in Figure 7, the calculated permittivity values are close to the average values *ε̅*.

## Conclusions

In this paper, we theoretically studied the permittivity of real polar liquids. These liquids differ greatly in the structure of their constituent molecules, as well as in the size, location, and orientation of their dipole moments. This diversity results in significantly different permittivity values observed for the polar liquids. Clearly, the permittivity reflects the polarizability of molecules and the dipole moments they carry. Can the dielectric constant be accurately estimated knowing the polarizability and dipole moment values of molecules, as well as some simple liquid characteristics, such as molar volume? To what extent will this description be universal?

Our study shows that a universal description of permittivity is indeed possible. This occurs when short-range correlations in the dipole system are negligible. In this case, permittivity becomes a function of the average dipole interactions, which are described by the effective Debye parameter *y*_eff_. This parameter is related to the polarizability and dipole moments of the molecules, as well as their concentration. The explicit universal expression [Disp-formula eq20] for *ε* is obtained from the third-order virial series in particle concentration, in which all terms depending on the coupling parameter λ are eliminated. The convergence of the series is limited by the condition *y*_eff_ ≪ 1. However, numerous experimental data on permeability of ferrofluids^7,8,13,20^ and the MC simulations for the dipolar HS system^21^ have demonstrated the applicability of eq 20 up to values of *y*_eff_ ≤ 2—see Figure 2. This surprising result allows us to apply the universal relation (eq 20) to real polar liquids.

The relationship was applied to approximately one hundred polar liquids with the permittivities *ε* ≤ 30. The main difficulty in the calculations was the values of the dipole moments of the molecules, which are unambiguously determined only in the gas phase. In solution, the magnitude of a molecule’s dipole moment depends on its neighbors. This dependence is especially evident in systems with hydrogen bonding.^44^ For polar liquids without hydrogen bonds, we use the dipole moment values determined in a benzene solution. For polar liquids with hydrogen bonds, we employ the values *m* obtained from numerical experiment. A comparison of the predicted and experimental values reveals very good agreement between them—see Figure 4. As can be seen, only about 15 liquids have noticeable differences in results. In four cases, this is due to the fact that the Debye parameter exceeds the threshold value, *y*_eff_ > 2. In six cases, the difference is most likely due to underestimated experimental values of the molecules’ dipole moments.

In Figure 5, we also consider an agreement between the theoretical predictions and the experimental data for the dipole moments of molecules. The former were derived from eq 20 and data on liquid permittivity. The agreement is so good that the relation [Disp-formula eq20] can serve as the simplest way to estimate the dipole moment of molecules with an approximate accuracy of ±0.1 D. In Figure 6, using three polar liquids as an example, we demonstrate that the universal description remains accurate over a fairly wide temperature range.

As we noted in the Introduction, the classical Onsager theory^27,28^ predicts underestimated permittivity values for liquids with *ε* ∼ 15–25, or *y*_eff_ = 1.5–2. For example, if in the case of 1-propanol we use the dipole moment value *m*_*p*_ = 1.56 D of pure liquid—see in Table 1—we obtain *ε*_Ons_ = 14.8 which is 40% less than the experimental value of 20.5. The difference would be almost 3-fold if the gas value *m*_*p*_ = 1.57 D were used instead, giving *ε*_Ons_ = 7.2. However, in the Onsager theory, the gas-phase values of *m* are assumed by default.^28^ For this reason, we do not perform a separate comparison of our and experimental results with the predictions of the Onsager model.

Finally, we generalized the eq 20 to the case of mixtures of polar liquids, assuming additivity of the mixture’s Debye parameter and volume. We verified the obtained relation [Disp-formula eq21] for several mixtures and determined that it provides an accurate lower estimate of the mixture permittivity. The slightest underestimation of the predictions occurs at a mole fraction of *x* ∼ 0.5, which can be attributed to the correlated arrangement of real mixture molecules.

## Supplementary Material



## Notes

A brief overview of the virial expansion of the dielectric constant of dipolar hard sphere fluid. Table S1 provides a list of the parameters for 101 polar liquids (PDF)
