# Supplementary material for: Predicting the Dielectric Constant of Real Polar Liquids
Source: ACS Omega. 2026 Aug 5;11(32):48515–25. doi: 10.1021/acsomega.6c05495 (PMC13750462; doi:10.1021/acsomega.6c05495)
Supplement: Supplementary file 1 [file ao6c05495_si_001.pdf]

Supporting Information

Predicting the Dielectric Constant of Real Polar Liquids

Konstantin I. Morozov*

Department of Chemical Engineering, Technion – Israel Institute of Technology, Haifa 32000, Israel

E-mail: mrk@technion.ac.il

Virial expansion for the dielectric constant of the dipolar hard sphere fluid

In this section, we will provide a brief overview of the virial expansion of the dielectric constant of dipolar hard sphere fluids, as discussed in.[1] We consider a system of dipolar hard spheres with a diameter of d and a permanent dipolar moment of m that interact via a pair potential:

$$U(1,2) = U_{\mathrm{HS}}(r) + U_d(1,2) = U_{\mathrm{HS}}(r) - (m^2/r^3)D(1,2)\,, \tag{S1}$$

where $U_{\mathrm{HS}}(r)$ is the hard sphere potential, $U_d(1,2)$ and $D(1,2)$ are the dipolar potential and its angular-dependent part, $D(1,2) = [3(\boldsymbol{e}_1\cdot\hat{\boldsymbol{r}})(\boldsymbol{e}_2\cdot\hat{\boldsymbol{r}}) - \boldsymbol{e}_1\cdot\boldsymbol{e}_2]$, $\boldsymbol{e}_i$ and $\hat{\boldsymbol{r}}$ are unit vectors along the dipolar moment of particle i, $\boldsymbol{m}_i = m\boldsymbol{e}_i$, and along the interparticle vector, $\boldsymbol{r}_{12} = r\hat{\boldsymbol{r}}$.

Depending on the dipole liquid sample's specific geometry, there are several ways to write its general permittivity expression in terms of the indirect correlation function, $h(1,2)$. Ref.[1] considered three formulations: the Debye, Onsager-Kirkwood, and Langevin formulations. The first formulation is obtained by examining a *spherical sample of dipole liquid in a vacuum.* The corresponding Debye-like relation for the dielectric constant ε is[1]

$$\frac{\varepsilon-1}{\varepsilon+2} = y\left[1 + \frac{n}{(4\pi)^2}\int h(1,2)\Delta(1,2)d\boldsymbol{e}_1 d\boldsymbol{e}_2 d\boldsymbol{r}_2\right]\,, \tag{S2}$$

where n is the number density, $y = 4\pi n m^2/(9k_BT)$ is the Debye parameter, $\Delta(1,2) = \boldsymbol{e}_1\cdot\boldsymbol{e}_2$ and the integration is performed over the orientations of two dipoles and their relative position.

The Onsager-Kirkwood form is followed for a *spherical sample embedded in a dielectric continuum with the same dielectric constant* ε

$$\frac{(\varepsilon-1)(2\varepsilon+1)}{9\varepsilon} = y\left[1 + \frac{n}{(4\pi)^2}\int h(1,2)\Delta(1,2)d\boldsymbol{e}_1 d\boldsymbol{e}_2 d\boldsymbol{r}_2\right]\,. \tag{S3}$$

Finally, Langevin formulation is specific to a *cylindrical sample, in which the weak external field is assumed to be parallel to the long axis of the cylinder*[1]

$$\varepsilon = 1 + 3y \left[1 + \frac{3n}{(4\pi)^2} \int h(1,2) e_{1,z} e_{2,z} d\boldsymbol{e}_1 d\boldsymbol{e}_2 d\boldsymbol{r}_2 \right] . \tag{S4}$$

Here $e_{1,z}$ and $e_{2,z}$ are the projections of the dipole moments onto the external field direction.

If the interparticle interactions are omitted, $h(1,2) = 0$, then the three equations (S2)-(S4) reduce to the standard one-particle models of Debye, Onsager and Langevin, respectively, – see Eqs. (3), (4) and (1) in the main text. When interparticle correlations are taken into account and $h(1,2) \neq 0$, all the approaches become equivalent. This is demonstrated directly in Ref.[1] in the lower orders of the virial expansion. It was also concluded that, of the three formulations of permittivity, the Langevin approach (S4) is best since it depends least on the approximations on the right-hand side of Eqs. (S2)-(S4).

The virial expansion is generated from Eq. (S4) after substitution of the density expansion of the correlation function $h(1,2)$[2]

$$h(1,2) = f(1,2) + \frac{n}{4\pi} \left[f(1,\mathbf{3}) f(\mathbf{3},2) + f(1,\mathbf{3}) f(\mathbf{3},2) f(1,2) \right] + \ldots , \tag{S5}$$

where $f(1,2) = \exp[-U(1,2)/k_B T] - 1$ is the Mayer function with the potential (S1) and the boldface is used for particle 3 to mark the integration over its coordinates. Relation (S5) is shown diagrammatically in Fig. S1 with obvious correspondence between the elements. The

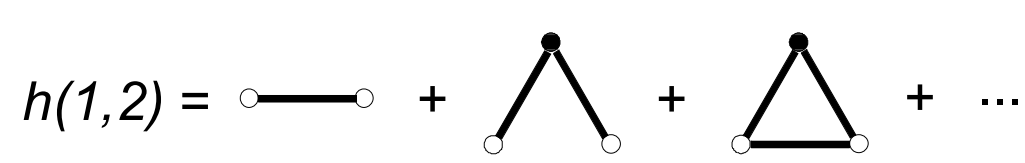

Figure S1: Diagram representation of the indirect correlation function $h(1,2)$.

second diagram with two lines relates to the class of reducible diagrams: its contribution into (S4) is factorized (see details in Ref.[1]). Substitution of the expansion (S5) into Langevin-like

relation (S4) yields after integration the virial series in the form

$$\varepsilon = 1 + 3y + 3y^2(1+b) + 3y^3(1+2b+b^2+c_\triangle) + \dots . \tag{S6}$$

Here b is the second virial coefficient and $c_\triangle$ is the contribution to the third virial coefficient related to the irreducible diagram with three lines in Fig. S1. Next the Mayer function $f(1,2)$ is decomposed over the powers of the dipolar potential $U_d(1,2) = -(m^2/r^3)D(1,2)$:

$$f(1,2) = f_{\mathrm{HS}}(r) + H(r-d)\sum_{k=1} \frac{1}{k!}\left[-\frac{m^2}{r^3}D(1,2)\right]^k , \tag{S7}$$

where $f_{\mathrm{HS}}(r)$ is the Mayer function of the hard sphere system and $H(x)$ is the Heaviside function taking nontrivial value $H(r-d) = 1$ when $r > d$. In the diagrammatic form this relation is

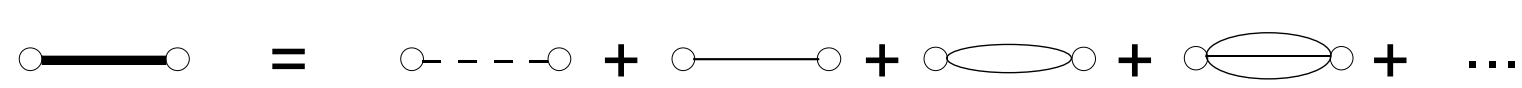

Figure S2: Diagram representation of the Mayer function $f(1,2)$ of the dipolar hard spheres via the Mayer function of the hard spheres, shown by the dashed line, and the dipole potential, U_d, marked by the thin solid lines. The dipolar lines are assumed to be taken at $r_{ij} > d$.

Substituting this representation into (S5) determines the entire virial series up to terms proportional to n^3. Note that single dipole lines lead to dependencies of ε on the long-range Debye parameter y, while dependence on the short-range parameter λ arises for multiple lines. The only irreducible diagram of the third virial coefficient that reduces to a constant is the Jepsen diagram, as shown on the left in Fig. S3:

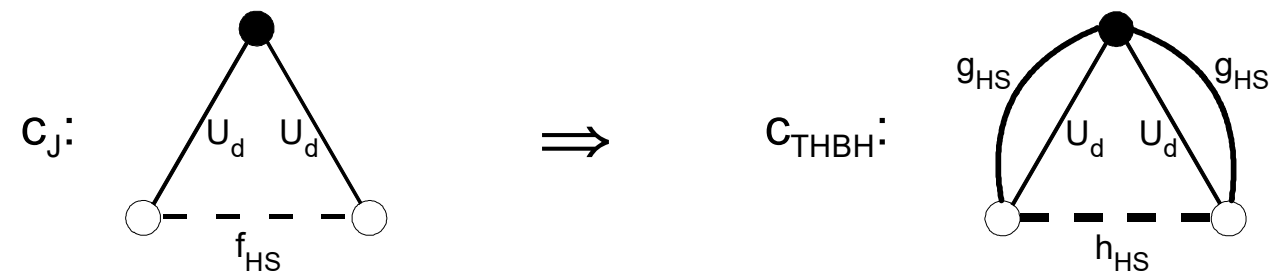

Figure S3: The Jepsen (left) and Tani-Henderson-Barker-Hecht (right) diagrams contribute to the third virial coefficients. The Jepsen diagram generates a λ-independent contribution to the third virial coefficient. In the dilute limits, the Tani-Henderson-Barker-Hecht diagram reduces to the Joslin one.

The explicit expression of the Jepsen contribution to the third virial coefficient is:[3]

$$c_J = \frac{3}{(4\pi)^2} \int D(1,3)D(3,2)H(r_{13}-d)H(r_{32}-d)f_{\mathrm{HS}}(r_{12})e_{1,z}e_{2,z}d\boldsymbol{e}_1 d\boldsymbol{e}_2 d\boldsymbol{r}_2 d\boldsymbol{r}_3 = -\frac{15}{16}. \quad \text{(S8)}$$

Finally, the third virial coefficient takes the form:

$$c = c_J + c_\lambda = -15/16 + c_\lambda\,. \quad \text{(S9)}$$

The function c_λ is calculated in Ref.[1] as a series on powers of λ and is indicated in the main text. Finally, the virial series (S6) of the permittivity has the following general form:

$$\varepsilon_{\mathrm{vir}} = 1 + 3y + 3y^2(1+b) + 3y^3(1/16 + 2b + b^2 + c_\lambda) + \ldots\,. \quad \text{(S10)}$$

However, in the approaches of Ivanov and Kuznetsova[4] and Tani-Henderson-Barker-Hecht,[5] some diagrams' contributions are omitted. In the former, all terms dependent on the parameter λ in the third virial coefficient are discarded, though they are retained in the latter.

$$\varepsilon_{\mathrm{IK}} = 1 + 3y + 3y^2(1+b) + 3y^3(1/16 + \xcancel{2b + b^2 + c_\lambda}) + \ldots\,, \quad \text{(S11)}$$

In Tani et al.'s approach,[5] all terms dependent on the parameter λ are omitted, and the contribution associated with the Jepsen diagram is modernized. Instead of the "bare" left diagram in Fig. S3, the "dressed" right version is considered. The pair distribution function

of hard spheres, $g_{\mathrm{HS}}(r)$, is inserted between vertices 1-3 and 2-3. The Mayer function, $f_{\mathrm{HS}}(r)$, is replaced by the indirect hard-sphere correlation function between vertices 1-2, $h_{\mathrm{HS}}(r)$:

$$\varepsilon_{\mathrm{THBH}} = 1 + 3y + 3y^2(1 + \cancel{b}) + 3y^3(1 + \xcancel{2b + b^2 + c_\lambda} + c_{\mathrm{THBH}}) + \ldots, \tag{S12}$$

In the main text, the full contribution to the third virial coefficient is denoted as $p = 1 + c_{\mathrm{THBH}}$ and it is indicated its Padé approximation (see Eq. (15)) found in Ref.[5] In the dilute limit, $p \to 1/16$.

List of liquid parameters

Table S1: List of liquid parameters. There are indicated the molar mass M, mass density ρ, refractive index $\sqrt{\varepsilon_\infty}$, dipole moment in solution and source of this value, effective Debye parameter y_{eff}, predicted ε_{uni} and experimental ε_{exp} values of the permittivity. The data for M, ρ, $\sqrt{\varepsilon_\infty}$ are taken from[6] at a temperature $T = 25°\text{C}$. The values of ε_{exp} were taken from.[6] These were obtained at $T = 25°\text{C}$, except for eight cases which are explicitly indicated by a superscript above the experimental value.

no	substance	M [g/mol]	ρ [g/cm^3]	$\sqrt{\varepsilon_\infty}$	m_l [D]	Ref.	y_{eff}	ε_{uni}	ε_{exp}
1	bromoethane	108.965	1.4500	1.4204	1.93	[7]	1.28	10.1	9.01
2	1-bromopropane	122.992	1.3451	1.4311	1.97	[7]	1.14	8.60	8.05
3	2-bromopropane	122.992	1.3031	1.4211	2.10	[8]	1.22	9.49	9.37
4	1-bromobutane	137.018	1.2686	1.4372	1.96	[7]	1.00	7.23	6.89
5	2-bromobutane	137.018	1.2524	1.4342	2.14	[7]	1.13	8.50	8.64
6	1-bromopentane	151.045	1.2119	1.4420	1.96	[7]	0.91	6.36	6.27
7	1-bromohexane	165.071	1.1679	1.4450	1.97	[7]	0.84	5.77	5.82
8	1-bromoheptane	179.098	1.1349	1.4479	1.97	[7]	0.79	5.30	5.33
9	2-bromoheptane	179.098	1.1227	1.4480	2.06	[9]	0.83	5.64	6.46[22]
10	3-bromoheptane	179.098	1.1349	1.4490	2.04	[9]	0.82	5.61	6.93[22]
11	4-bromoheptane	179.098	1.1300	1.4472	2.04	[9]	0.82	5.61	6.81[22]
12	bromobenzene	157.010	1.4886	1.5568	1.55	[8]	0.82	5.54	5.40
13	o-bromotoluene	171.035	1.4166	1.5534	1.45	[8]	0.70	4.66	4.57
14	m-bromotoluene	171.035	1.4033	1.5479	1.77	[8]	0.87	6.00	5.48
15	p-bromotoluene	171.035	1.4092	1.5446	1.90	[10]	0.95	6.72	6.0[27.2, a]
16	chloromethane	50.488	0.9110	1.3360	1.69	[13]	1.27	10.0	9.77
17	chloroethane	64.514	0.8902	1.3651	1.89	[14]	1.24	9.71	9.32
18	1-chloropropane	78.541	0.8866	1.3863	1.93	[14]	1.11	8.23	8.36
19	2-chloropropane	78.541	0.8585	1.3761	2.08	[8]	1.21	9.31	9.52
20	1-chlorobutane	92.567	0.8803	1.3995	1.92	[14]	0.97	6.91	7.09
21	2-chlorobutane	92.567	0.8678	1.3943	2.10	[8]	1.10	8.14	8.39
22	1-chloropentane	106.594	0.8771	1.4100	1.94	[14]	0.89	6.21	6.50

[a] The melting point of p-bromotoluene (p-btl) is 26.2°C. The temperature dependence of its permittivity ε_{exp} is indicated in[6] only for solid phase. The only measurement of the dielectric constant of p-bromotoluene in the liquid phase at $T = 27.2°\text{C}$ that we know of was made by Roberts.[11] He obtained the value $\varepsilon_{\text{exp,p-btl}} = 6.0$. However, the value 8.3, which was found for methyl nonyl ketone in the *adjacent* row of Table II of Ref.[11] and included in a number of handbooks[12] and electronic databases, was mistakenly used instead.

no	substance	M [g/mol]	ρ [g/cm^3]	$\sqrt{\varepsilon_\infty}$	m_l [D]	Ref.	y_{eff}	ε_{uni}	ε_{exp}
23	1-chlorohexane	120.62	0.8728	1.4171	1.97	[14]	0.84	5.74	5.95
24	1-chloroheptane	134.647	0.8719	1.4240	1.97	[14]	0.78	5.27	5.39
25	2-chloroheptane	134.647	0.8629	1.4197	2.03	[9]	0.81	5.47	6.52[22]
26	3-chloroheptane	134.647	0.8647	1.4204	2.04	[9]	0.81	5.52	6.70[22]
27	4-chloroheptane	134.647	0.8667	1.4213	2.04	[9]	0.81	5.54	6.54[22]
28	chlorobenzene	112.557	1.1002	1.5209	1.58	[8]	0.83	5.64	5.70
29	o-chlorotoluene	126.584	1.0778	1.5240	1.40	[8]	0.67	4.42	4.64
30	m-chlorotoluene	126.584	1.0703	1.5187	1.75	[8]	0.86	5.88	5.66
31	p-chlorotoluene	126.584	1.0632	1.5123	1.90	[8]	0.94	6.65	6.19
32	fluorobenzene	96.102	1.0191	1.4594	1.45	[8]	0.75	4.99	5.42
33	tetrahydrofuran	72.106	0.8833	1.4050	1.75	[8]	1.02	7.42	7.43
34	tetrahydropyran	86.132	0.8761	1.4171	1.68	[8]	0.85	5.85	5.61
35	methyl acetate	74.079	0.9277	1.3586	1.76	[15]	1.02	7.40	6.86
36	ethyl acetate	88.106	0.8944	1.3695	1.84	[15]	0.94	6.61	5.99
37	propyl acetate	102.132	0.8824	1.3816	1.79	[10]	0.81	5.49	5.52
38	sec-butyl acetate	116.158	0.8694	1.3861	1.86	[10]	0.77	5.21	5.04
39	tert-butyl acetate	116.158	0.8615	1.3830	1.92	[10]	0.80	5.43	5.51
40	pentyl acetate	130.185	0.8710	1.3999	1.90	[8]	0.75	5.00	4.73
41	methyl ethyl ketone	72.106	0.7999	1.3760	2.76	[8]	1.97	19.9	18.3
42	methyl propyl ketone	86.132	0.8041	1.3869	2.77	[10]	1.71	15.8	15.3
43	diethyl ketone	86.132	0.8098	1.3905	2.82	[10]	1.78	16.9	16.8
44	2-hexanone	100.158	0.8068	1.3982	2.73	[16]	1.48	12.6	14.2
45	2-heptanone	114.185	0.8068	1.4063	2.71	[16]	1.32	10.6	11.7
46	3-heptanone	114.185	0.8139	1.4032	2.81	[8]	1.41	11.7	12.4
47	4-heptanone	114.185	0.8131	1.4044	2.66	[16]	1.29	10.2	12.3
48	1,3-dioxane	88.106	1.0286	1.4165	2.15	[8]	1.37	11.2	13.6
49	1,4-dioxane	88.106	1.0337	1.4224	0.45	[8]	0.31	2.25	2.21
50	dichloromethane	84.933	1.3170	1.4207	1.55	[13]	1.03	7.46	8.93
51	dibromomethane	173.835	2.4842	1.5387	1.41	[17]	0.92	6.44	7.23
52	diiodomethane	267.836	3.3092	1.7376	1.22	[10]	0.83	5.69	5.32
53	1,1-dichloroethane	98.959	1.1679	1.4132	2.00	[8]	1.23	9.55	10.1
54	1,2-dibromoethane	187.861	2.1683	1.4422	1.20	[8]	0.68	4.46	4.78
55	1,2-dichloropropane	112.986	1.1494	1.465	1.87	[8]	1.00	7.22	8.21
56	1,3-dichloropropane	112.986	1.1687	1.4430	2.15	[8]	1.26	9.89	10.5
57	2,2-dichloropropane	112.986	1.1067	1.4119	2.20	[8]	1.23	9.58	10.9
58	1,2-dibromopropane	201.888	1.9248	1.5177	1.43	[18]	0.72	4.82	4.55
59	1,3-dibromopropane	201.888	1.9627	1.5180	2.05	[8]	1.16	8.82	9.29

no	substance	M [g/mol]	ρ [g/cm^3]	$\sqrt{\varepsilon_\infty}$	m_l [D]	Ref.	y_{eff}	ε_{uni}	ε_{exp}
60	o-dichlorobenzene	147.002	1.3002	1.5456	2.27	[8]	1.27	10.1	10.0
61	m-dichlorobenzene	147.002	1.2827	1.5409	1.50	[8]	0.74	4.95	4.8
62	o-dibromobenzene	235.904	1.9754	1.6121	2.13	[8]	1.16	8.80	7.81
63	m-dibromobenzene	235.904	1.9455	1.6057	1.50	[8]	0.76	5.08	4.76
64	pyridine	79.101	0.9765	1.5062	2.20	[8]	1.54	13.4	13.0
65	diethyl malonate	160.168	1.0498	1.4115	2.54	[10]	1.13	8.44	7.65
66	methyl benzoate	136.15	1.0837	1.5140	1.92	[17]	0.92	6.48	6.74
67	ethyl benzoate	150.174	1.0415	1.4980	2.00	[15]	0.88	6.10	6.10
68	trichloromethane	119.378	1.4788	1.4435	1.13	[13]	0.60	3.95	4.71
69	tribromomethane	252.731	2.8788	1.5948	1.02	[17]	0.61	4.02	4.25
70	anisole	108.138	0.9894	1.5146	1.25	[8]	0.62	4.03	4.23
71	methyl methacrylate	100.117	0.9377	1.4118	1.78	[19]	0.87	5.99	6.45
72	ethyl methacrylate	114.142	0.9083	1.4120	1.85	[19]	0.82	5.57	5.81
73	iodomethane	141.939	2.2645	1.5268	1.41	[20]	0.98	6.99	6.87
74	iodoethane	155.965	1.9239	1.5096	1.70	[20]	1.05	7.67	7.62
75	1-iodopropane	169.992	1.7392	1.5025	1.88	[8]	1.06	7.73	6.97
76	2-iodopropane	169.992	1.6944	1.4994	2.00	[8]	1.13	8.49	8.19
77	1-iodobutane	184.018	1.6068	1.4970	1.90	[8]	0.96	6.79	6.18
78	2-iodobutane	184.018	1.5835	1.4960	2.10	[8]	1.09	8.06	7.78
79	diethyl ether	74.121	0.7079	1.3491	1.30	[8]	0.55	3.61	4.24
80	dibutyl ether	130.228	0.7641	1.3967	1.20	[8]	0.42	2.83	3.05
81	o-chloroaniline	127.572	1.2130	1.5870	1.75	[21]	0.96	6.84	6.90
82	m-chloroaniline	127.572	1.2107	1.5908	2.40	[21]	1.49	12.7	12.3
83	cyclohexanone	98.142	0.9435	1.4484	3.08	[8]	2.15	23.1	15.6
84	acetone	58.079	0.7845	1.3560	2.77	[8]	2.35	27.0	20.5
85	ethanethiol	62.134	0.8315	1.4285	1.48	[8]	0.87	6.01	6.67
86	1-propanethiol	76.161	0.8362	1.4351	1.51	[8]	0.79	5.31	5.77
87	2-propanethiol	76.161	0.8090	1.4223	1.60	[8]	0.82	5.61	5.95
88	2-methylpyridine	93.127	0.9396	1.4928	2.04	[8]	1.17	8.91	9.96
89	3-methylpyridine	93.127	0.9520	1.5011	2.40	[8]	1.51	13.1	11.7
90	4-methylpyridine	93.127	0.9503	1.5009	2.58	[8]	1.70	15.7	12.0
91	divinyl ether	70.09	0.7666	1.3951	1.07	[8]	0.51	3.32	3.94[15]
92	propylamine	59.110	0.7122	1.3839	1.35	[8]	0.69	4.58	5.00
93	diethylamine	73.137	0.7004	1.3832	1.14	[8]	0.50	3.26	3.58
94	trichloroethylene	131.388	1.4561	1.4742	0.95	[8]	0.50	3.30	3.42
95	ethoxyacetylene	70.090	0.7948	1.3768	1.98	[8]	1.15	8.69	8.05
96	phosphorus oxychloride	153.331	1.645	1.46	2.4	[8]	1.55	13.6	14.1
97	isopropylamine	59.110	0.6836	1.3708	1.45	[10]	0.73	4.89	5.51

no	substance	M [g/mol]	ρ [g/cm^3]	$\sqrt{\varepsilon_\infty}$	m_l [D]	Ref.	y_{eff}	ε_{uni}	ε_{exp}
98	ethanol	46.068	0.7849	1.3590	1.73	[8]	1.27	10.1	24.9[a]
					2.56	[22]	2.51	30.4	24.9
99	1-propanol	60.095	0.7997	1.3837	1.70	[8]	1.03	7.48	20.5
					2.56	[22]	2.03	21.0	20.5
100	2-propanol	60.095	0.7809	1.3750	1.70	[8]	1.01	7.25	19.3
					2.71	[22]	2.19	24.0	19.3
101	tert-butanol	74.121	0.7750	1.3878	1.70	[8]	0.86	5.96	12.2
					2.41	[22]	1.29	12.7	12.2

[a] For four alcohols, the second (lower) value of the dipole moment is given for the pure liquid.

References

(1) Morozov, K. I. The dielectric virial expansion and the models of dipolar hard-sphere fluid. *J. Chem. Phys.* **2007**, *126*, 194506.

(2) Hansen, J.-P.; McDonald, I. R. *Theory of Simple Liquids*, 2nd ed. Academic, London, 1990.

(3) Jepsen, D. W. Calculation of the dielectric constant of a fluid by cluster expansion methods. *J. Chem. Phys.* **1966**, *44*, 774–781.

(4) Ivanov, A. O.; Kuznetsova, O. B. Magnetic properties of dense ferrofluids: An influence of interparticle correlations. *Phys. Rev. E* **2001**, *64*, 041405.

(5) Tani, A.; Henderson, D.; Barker, J. A.; Hecht, C. E. Application of perturbation theory to the calculation of the dielectric constant of a dipolar hard sphere fluid. *Mol. Phys.* **1983**, *48*, 863–869.

(6) Lide, D. R. Ed. *CRC Handbook of Chemistry and Physics*, 84th ed.; CRC Press: New York, 2003–2004.

(7) Le Fèvre, R. J. W.; Williams, A. J. 768. The polarisations and apparent dipole moments of fourteen n-alkyl bromides between methyl and octadecyl in carbon Tetrachloride. *J. Chem. Soc.* **1965**, *0*, 4185–4188.

(8) McClellan, A. L. *Tables of experimental dipole moments*; W. H. Freeman: San Francisco, 1963.

(9) Errera, J.; Sherrill, M. L. An investigation of a series of derivatives of normal heptane. II. Dipole moments and volecular constitution. *J. Am. Chem. Soc.* **1930**, *52*, 1993–1997.

(10) Yaws, C. L.; Narasimhan, P. K. *Dipole moment – organic compounds.* In: Carl L. Yaws, C. L. Ed. Thermophysical Properties of Chemicals and Hydrocarbons, William Andrew Publishing, 2009, pp. 672-682.

(11) Roberts, G. N. The dielectric properties of supercooled liquids. *J. Am. Chem. Soc.* **1953**, *75*, 2264–2266.

(12) Wohlfahrt, Ch. *2 Pure Liquids: Data: Datasheet from Landolt-Börnstein - Group IV Physical Chemistry · Volume 6: "Static Dielectric Constants of Pure Liquids and Binary Liquid Mixtures", Edt. Madelung, O.*; Springer-Verlag: Berlin, 1991. DOI: 10.1007/10047452_2.

(13) Barclay, G. A.; Le Fèvre, R. J. W. 105. The apparent dipole moments of the chlorinated methanes in the liquid, dissolved, and gaseous states. *J. Chem. Soc.* **1950**, *0*, 556–562.

(14) Le Fèvre, R. J. W.; Orr, B. J. Molecular polarisability and internal rotation in the alkyl chloride series. *J. Chem. Soc. B: Phys. Org.* **1966**, *0*, 37–40.

(15) Pinkus, A. G.; Lin, E. Y. Electric dipole moment studies of carboxylic ester conformations: alkyl acetates, benzoates, 2, 4, 6-trimethyl-benzoates and 2, 3, 5, 6-tetramethylbenzoates. *J. Mol. Struct.* **1975**, *24*, 9–26.

(16) Vij, J. K.; Hufnagel, F. Dielectric relaxation and libration spectroscopy of some aliphatic ketones and their molecular behavior. *J. Phys. Chem.* **1991**, *95*, 6142–6148.

(17) Buckingham, A. D.; Le Fèvre, R. J. W. 687. A comparison of the apprent dipole moments of the brominated methanes dissolved in benzene with the true values determined as gases. *J. Chem. Soc.* **1953**, *0*, 3432–3435.

(18) Thorbjørnstrud, J.; Ellestad, O. H.; Klaboe, P.; Torgrimsen, T. V. The vibrational spectra, dipole moments and conformations of 1,2-dichloro-, 1-chloro-2-bromo- and 1,2-dibromopropane. *J. Mol. Struct.* **1973**, *15*, 45–59.

(19) Mikhailov, G. P. Dielectric losses and polarization of polymers. *J. Polym. Sci.* **1958**, *30*, 605–614.

(20) Krishna, B.; Srivastava, K. K. Experimental and theoretical aspects of Higasi's equation for a quick determination of electric dipole moment in solution. *J. Phys. Chem.* **1960**, *32*, 663–664.

(21) Kadam, S. S.; Hudge, P. G.; Suryawanshi, D. B.; Kumbharkhane, A. C. Dielectric relaxation study of 2 and 3-chloroaniline and 2 and 3-methoxyaniline with1,4-dioxane mixtures using time domain technique. *J. Mol. Liq.* **2013**, *177*, 426–431.

(22) Jorge, M.; Gomes, J. R. B.; Barrera, M. C. The dipole moment of alcohols in the liquid phase and in solution. *J. Mol. Liq.* **2022**, *356*, 119033.
